# Virtual screening and MD simulation-guided discovery of CDK12-IN-3, a potent ALK inhibitor suppressing neuroblastoma growth *in vitro* and *in vivo*

**DOI:** 10.3389/fchem.2026.1870293

**Published:** 2026-08-10

**Authors:** Tianyi Liu, Xuejiao Hu, Wenxin Yan, Hongli Yin, Zhong Li

**Affiliations:** 1 Department of Pharmacy, Dalian Women and Children’s Medical Group, Dalian, Liaoning, China; 2 Department of Pharmacy, Women and Children’s Hospital of Dalian University of Technology, Dalian, Liaoning, China; 3 College of Pharmacy, Dalian Medical University, Dalian, Liaoning, China; 4 Institute of Pediatric Research, Children’s Hospital of Soochow University, Suzhou, China

**Keywords:** ALK, CDK12-IN-3, molecular dynamics simulation, neuroblastoma, virtual screening

## Abstract

Neuroblastoma (NB) is the most common extracranial malignant solid tumor in children, and aberrant activation of anaplastic lymphoma kinase (ALK)—driven primarily by hotspot mutations F1174L and R1275Q—represents a core oncogenic driver of high-risk and relapsed NB. Clinical ALK inhibitors are frequently hampered by acquired drug resistance and off-target toxicity, highlighting an unmet need for novel selective ALK inhibitors for NB treatment. Here, we employed a drug repurposing strategy integrating virtual screening, multi-dimensional molecular dynamics (MD) simulations, steered MD, and umbrella sampling to screen 7,322 compounds for novel ALK inhibitory activity, followed by systematic *in vitro* and *in vivo* validation. We identified CDK12-IN-3 (C18) as a high-affinity ligand for wild-type ALK and both NB-associated ALK mutants (F1174L, R1275Q), with favorable binding selectivity over 10 homologous tyrosine kinases. *In vitro*, CDK12-IN-3 exerted nanomolar-level antiproliferative, anti-clonogenic, and anti-migratory effects across three genetically distinct NB cell lines (SH-SY5Y, SK-N-BE (2), IMR-32), while showing no significant cytotoxicity in ALK-negative HEK293T normal cells even at 20 μM, demonstrating a wide *in vitro* safety window. Mechanistic studies confirmed that CDK12-IN-3 directly bound ALK (KD = 5.33 μM by surface plasmon resonance), downregulated ALK expression, suppressed the downstream PI3K/AKT/mTOR signaling pathway, and induced NB cell apoptosis. *In vivo*, oral administration of CDK12-IN-3 (50 mg/kg) significantly suppressed NB xenograft tumor growth in nude mice with no observable systemic toxicity. Collectively, our findings validate CDK12-IN-3 as a novel selective ALK inhibitor with potent anti-NB activity, providing a promising lead compound and an efficient dynamic simulation-guided drug repurposing paradigm for overcoming ALK inhibitor resistance in high-risk NB.

## 1 Introduction

Neuroblastoma (NB) arises from primitive neuroepithelial cells of the embryonic neural crest and develops along the sympathetic nervous system, representing the most common extracranial malignant solid tumor in children (Sainero-Alcolado et al., 2024). Currently, clinical practice adopts multimodal comprehensive therapy based on the International Neuroblastoma Risk Group (INRG) risk stratification: low-risk patients are primarily managed with observation or minimal therapy, and some cases may undergo spontaneous regression; high-risk patients require intensive therapy, the core components of which include multi-drug induction chemotherapy, surgery, radiotherapy, high-dose chemotherapy combined with autologous stem cell transplantation, and anti-GD2 monoclonal antibody immunotherapy, among others (Pieniążek et al., 2024). Conventional therapies have notable limitations. Chemotherapy frequently induces systemic toxicity and drug resistance; surgery carries a high risk of complications and residual lesions. In pediatric patients, radiotherapy damages normal tissues and increases the risk of growth retardation and secondary malignancies. Autologous stem cell transplantation involves complex procedures, high costs, and considerable recurrence risk, while anti-GD2 monoclonal antibodies often trigger severe adverse reactions that impair treatment compliance (Del Bufalo et al., 2023; Stip et al., 2023). Small-molecule targeted drugs act precisely on tumor-specific targets, exhibiting the advantages of highly effective tumor suppression and minimal damage to normal tissues, with milder toxic and side effects and more definitive efficacy—thus offering a new direction for improving the prognosis of pediatric NB patients.

Anaplastic Lymphoma Kinase (ALK) acts as a key oncogenic driver in NB, and its aberrant activation coupled with dysregulated upstream and downstream signal regulation plays a central role in tumor initiation and progression—particularly serving as a definitive etiological driver in familial NB and high-risk sporadic cases. Aberrant activation of ALK is predominantly mediated by point mutations, with an overall incidence of 6%–12% in NB patients, rising to 14% in high-risk cohorts and being even more markedly elevated in relapsed patients (Aktaş et al., 2023; Alam et al., 2019). These mutations are concentrated primarily in hotspot loci within the tyrosine kinase domain, such as F1174 (e.g., F1174L/V) and R1275 (e.g., R1275Q), which confer ligand-independent constitutive activation of ALK (Aktaş et al., 2023; Kwon et al., 2011). Additionally, gene amplification, ligand-dependent activation (e.g., via ALKAL1/2), and the formation of rare fusion proteins (e.g., TENM3-ALK) can also induce ALK activation (Durand et al., 2019). Notably, ALK mutation status is closely associated with poor prognosis in intermediate and high-risk patients (Aktaş et al., 2023), and heterogeneous mutation profiles may be observed across distinct anatomical sites (e.g., primary lesions vs. bone marrow metastases) in the same patient, indicative of clonal evolution (Durand et al., 2019). Aberrantly activated ALK drives the malignant phenotype of NB through multiple signaling pathways. The RAS/MAPK pathway promotes proliferation and survival via the RAS-RAF-MEK-ERK cascade (Berlak et al., 2022). The PI3K/AKT/mTOR pathway inhibits apoptosis and enhances protein synthesis through PIP3-AKT signaling (Sampson et al., 2024). The JAK/STAT pathway activates STAT3 to upregulate anti-apoptotic genes and potentiate tumor invasion (Qin et al., 2024). More critically, ALK forms a positive feedback regulatory loop with MYCN, a core oncogenic factor in NB—ALK directly binds to the MYCN promoter to enhance its transcription and upregulate MYCN expression (Tucker et al., 2019), while MYCN in turn binds to the ALK promoter to augment ALK expression, with the two synergistically amplifying oncogenic effects (Parkinson et al., 2022).

As ALK is a key oncogenic driver in NB, targeted ALK intervention has become a research hotspot, offering a promising direction for improving outcomes in high-risk and relapsed patients. Currently, ALK inhibitors have formed a well-defined multi-generation clinical application system, with core drugs including first-generation Crizotinib, second-generation Ceritinib and Alectinib, as well as third-generation Lorlatinib. These agents have demonstrated excellent initial efficacy in the treatment of ALK-positive tumors (including non-small cell lung cancer [NSCLC], NB, etc.), bringing significant survival benefits to patients. However, an unavoidable dilemma in clinical practice is that nearly all patients eventually develop drug resistance, and the resistance mechanisms exhibit high heterogeneity (Katayama, 2018). ALK-dependent resistance (on-target resistance) is the most core mechanism, primarily driven by mutations in the ALK kinase domain. To date, nearly 20 mutation types have been identified, and the selective pressure exerted by different inhibitors induces characteristic mutation profiles—for instance, Crizotinib resistance is dominated by L1196M (gatekeeper mutation) and C1156Y (Song et al., 2015); Alectinib resistance commonly involves mutations such as G1202R and I1171N (Shi et al., 2020); while third-generation Lorlatinib resistance is mostly characterized by compound mutations like G1202R + L1196M (Shaw et al., 2019). Moreover, the number of mutations detected in patients after treatment with second-generation tyrosine kinase inhibitors (TKIs) is significantly higher than that after first-generation drug therapy (Berlak et al., 2022). In addition, mechanisms such as bypass activation, phenotypic alterations, and overexpression of drug efflux pumps also contribute to the development of resistance, further increasing the difficulty of treatment. Therefore, the development of a new generation of ALK inhibitors with broad-spectrum anti-mutation activity has become a core demand to break through the limitations of current therapies.

Despite these advances, current ALK inhibitors face significant challenges in the NB context. Lorlatinib, the most potent third-generation ALK inhibitor, demonstrated clinical activity across all NB-associated ALK hotspot mutations (F1174L, R1275Q, F1245C) in a first-in-child phase 1 trial; however, the objective response rate as monotherapy in pediatric patients was only 30% (modified response criteria), with 27% of patients developing off-target resistance via RAS-MAPK pathway mutations and 15% acquiring on-target compound ALK mutations (e.g., F1174L/G1202R, F1174L/L1196M) (Berko et al., 2023; Goldsmith et al., 2023). Moreover, lorlatinib treatment is associated with a substantial toxicity burden, including hypertriglyceridemia (90%), weight gain (87%), and hypercholesterolemia (79%), and pediatric patients require approximately twice the adult dose to achieve therapeutic exposure (Roskoski, 2024). Brigatinib, a second-generation ALK inhibitor with approximately 12-fold greater potency than crizotinib, has shown preclinical efficacy in NB models (Zhang et al., 2016) but lacks clinical data in the pediatric NB setting. Entrectinib, a pan-ALK/TRKA/B/C/ROS1 inhibitor, offers broader target coverage but at the cost of reduced selectivity, raising concerns about off-target toxicity in the pediatric population (Johannes et al., 2018). Critically, all currently approved ALK inhibitors function exclusively as ATP-competitive kinase inhibitors and do not affect ALK protein expression levels, leaving the compensatory reactivation of ALK-mediated downstream signaling as a persistent resistance mechanism. These limitations underscore the urgent need for ALK inhibitors with novel structural scaffolds, dual mechanisms of action, and improved safety profiles for NB treatment.

To address the unmet needs for targeted therapy of ALK-driven NB and circumvent the limitations of traditional new drug development (e.g., long development cycles and high costs), this study adopted drug repurposing—a highly efficient research and development strategy—and integrated virtual screening with multi-dimensional molecular dynamics simulation techniques to establish a comprehensive research framework spanning target optimization, compound screening, and activity validation (Wang et al., 2026). As an innovative approach in the field of tumor targeted therapy in recent years, drug repurposing has been proven to be highly efficient in various cancer studies: by exploring new indications for existing compounds, it can significantly shorten the research and development cycle and reduce costs, making it particularly suitable for therapeutic research on rare diseases such as NB.

Through an innovative technical workflow, this study confirmed that CDK12-IN-3 exhibits potential ALK inhibitory activity via virtual screening combined with molecular dynamics (MD), steered molecular dynamics (SMD), and umbrella sampling (US) techniques. Further validation by *in vitro* and *in vivo* functional assays demonstrated that CDK12-IN-3 potently inhibits the proliferation, migration, and clonogenic formation capacity of NB cells *in vitro*; meanwhile, it downregulates the expression level of ALK, suppresses the activation of the downstream PI3K/AKT signaling pathway, and induces cell apoptosis without significant off-target effects. *In vivo* experiments using xenograft tumor models in nude mice also indicated that this compound exerts remarkable anti-tumor activity with no obvious toxicity. Based on the drug repurposing strategy, CDK12-IN-3 identified and validated through multi-dimensional technical screening in this study provides a novel insight and important experimental evidence for resolving the drug resistance mediated by ALK mutations and optimizing the therapeutic strategy for high-risk NB.

## 2 Methods and materials

### 2.1 Virtual screening

#### 2.1.1 ALK protein target structure preprocessing

In this study, the wild-type ALK protein (PDB ID: 4MKC) and F1174L mutant ALK protein (PDB ID: 2YJR) were first retrieved and downloaded from the Protein Data Bank (PDB) (Epstein et al., 2012; Friboulet et al., 2014). ALK structures were preprocessed using the Protein Preparation module of Schrödinger Maestro 13.5. After importing the PDB files, non-essential water molecules, cocrystallized ligands, and free ions (Cl^−^, Na^+^) were removed, while structurally important water molecules mediating receptor-ligand interactions were retained. Bond order correction and formal charge assignment were completed based on the software’s default OPLS4 force field, and abnormal amino acid residues were automatically identified and corrected synchronously (e.g., configuration correction of isomerized proline, reductive repair of oxidized methionine, etc.). The missing structural regions of the proteins were complemented via the Prime module integrated in the software to ensure structural continuity. The protonation states of key amino acids were precisely optimized according to the physiological environment at pH 7.4. Energy minimization was performed to further guarantee the high accuracy and reliability of the protein structures. Upon completion of structural preprocessing, the ALK inhibitor binding pocket was defined using the “Protein Grid Generation” module of Maestro 13.5: With reference to published structure-activity relationship data of ALK inhibitors, the molecular centroid of the positive control drug AZD-3463 (Wang et al., 2016) was set as the origin, and a cubic molecular docking box with an edge length of 20 Å was constructed for grid configuration in subsequent virtual screening of ALK inhibitors.

#### 2.1.2 Preprocessing of ligand databases

To meet the research and development requirements for ALK kinase inhibitors, this study established an integrated screening system incorporating four compound libraries with complementary functions: the MCE Kinase Inhibitor Library (1,513 compounds, official access: https://www.medchemexpress.com/screening/Kinase_Inhibitor_Library.html), which supplies potential lead compounds targeting the ALK kinase domain; the APExBIO Anticancer Compound Library (1,777 compounds, official access: https://www.apexbt.com/discoveryprobetm-anti-cancer-compound-library.html) encompassing anticancer molecules with diverse mechanisms of action; the Plus Preclinical and Clinical Compound Library (965 compounds, official access: https://www.apexbt.com/bioactive-compound-library-plus.html) that acts as a pivotal bridge connecting preclinical candidate drugs and clinical-stage molecules; and the L1300-FDA Approved Drug Library (3,067 compounds, official access: https://www.selleckchem.com/screening/fda-approved-drug-library.html), which capitalizes on their well-defined safety characteristics to broaden the potential for repurposing approved pharmaceuticals. Collectively, these four compound libraries comprise 7,322 compounds, and the research team carried out structural preprocessing on them using the LigPrep module of Maestro 13.5 software. The specific operations are as follows: protonation modification under physiological pH conditions and desalination to eliminate counterions; hydrogen atom addition and tautomer generation to account for potential structural variations of molecules; determination of stereochemical configurations to ensure structural precision; and geometric optimization via the OPLS4 force field to endow compounds with conformational stability. Subsequently, the research team implemented multi-dimensional druggability screening through the QikProp module of Maestro 13.5 software, excluding molecules with unsatisfactory pharmacokinetic properties. Ultimately, 18,005 conformationally stable candidate compounds were obtained, all of which satisfy the druggability assessment criteria and are applicable to subsequent virtual screening procedures.

#### 2.1.3 Virtual screening

In the first screening phase, a three-dimensional pharmacophoric feature field of the target was constructed based on the OPLS4 force field, and high-throughput primary screening of compounds was conducted via the Glide HTVS (High-Throughput Virtual Screening) mode, with the top 30% of candidate molecules ranked by GlideScore retained. In the second phase, the all-atom Standard Precision (SP) docking algorithm was employed to analyze the backbone cooperative motion of ligand-receptor complexes; after energy optimization, the top 30% of compounds were selected to proceed to the Extra Precision (XP) docking phase. In the final phase, induced-fit flexible docking was performed, and the binding free energy (ΔGbind) was calculated in combination with the MM/GBSA implicit solvent model, thereby screening out potential high-affinity ligands targeting ALK. To validate the docking protocol, the co-crystallized ligands of ALK (PDB: 4MKC) and ALK (F1174L) (PDB: 2YJR) were re-docked into their respective binding pockets using the same Glide docking protocol. The root-mean-square deviation (RMSD) of non-hydrogen atoms between the docked and experimental poses was less than 2.0 Å, confirming the reliability of the docking workflow. Key ADME pharmacokinetic parameters of the hit compounds were predicted using the SwissADME online server (https://swissadme.ch/). Potential toxicity risks of the candidate compounds were evaluated via the ProTox 3.0 web platform (https://tox-new.charite.de/protox3/) (Muthuvairam Subbulakshmi et al., 2026).

#### 2.1.4 DFT calculations

To evaluate the structural stability and electronic properties of the hit compounds prior to MD simulations, density functional theory (DFT) calculations were performed using Gaussian 16W (Gaussian, Inc., Wallingford, CT, United States). Geometry optimization and frequency analysis were carried out at the B3LYP/6-31+G (d′) level of theory. The calculated parameters included SCF energy, zero-point energy-corrected energy (EZPE-corr), Gibbs free energy at 298 K (G298K), dipole moment, and Frontier molecular orbital energies (HOMO, LUMO, and HOMO–LUMO gap) (Saha and Kawsar, 2025).

### 2.2 MD simulation

Referring to the methods of previous studies (Liu et al., 2024), the complex system was constructed using GROMACS 2023.1 with the optimal conformation from virtual screening as the initial structure: the protein was parameterized with the AMBER99SB-ILDN force field, and ligand topology files were generated using the AMBER force field with AM1-BCC partial charges via Acpype. A water box was constructed using the TIP3P water model, and Na^+^ and Cl^−^ were added to neutralize the system charge. Energy optimization was performed with the steepest descent algorithm, followed by NVT equilibration (310 K, V-rescale thermostat, 100 ps) and NPT equilibration (1 atm, Parrinello-Rahman barostat, 100 ps) in sequence. 100 ns MD simulations were conducted (a trajectory frame saved every 10 ps, independently repeated 5 times), and periodic boundary conditions were applied to eliminate boundary effects. Binding free energies (ΔG_bind_) were calculated using the MM/GBSA method implemented in gmx_MMPBSA 1.6.1. For each 100 ns trajectory (10,000 frames saved every 10 ps), the first 5,000 frames (0–50 ns, equilibration phase) were discarded and frames 5,001–10,000 (50–100 ns, equilibrium phase) were used for energy calculation. The generalized Born (GB) implicit solvent model was employed with igb = 5 (OBC2 model) and an ionic strength of 0.15 M NaCl. The binding free energy was computed as ΔGbind = ΔE_MM (ΔE_vdW + ΔE_elec) + ΔG_solv, where ΔG_solv comprises the polar solvation energy (ΔG_GB, calculated by the GB model) and the non-polar solvation energy (ΔG_SA, calculated by the SASA model). Conformational entropy contribution (−TΔS) was not included. All other parameters used default settings. Trajectory convergence was assessed by monitoring the backbone RMSD; a trajectory was considered converged when the RMSD fluctuation remained within 0.2 nm over the equilibrium phase (50–100 ns). The backbone RMSD of ALK and the ligand RMSD of non-hydrogen atoms in hit compounds were calculated. Trajectory convergence was assessed by monitoring the backbone RMSD; a trajectory was considered converged when the RMSD fluctuation remained within 0.2 nm over the equilibrium phase (50–100 ns). Free energy landscapes (FELs) were further constructed using protein backbone RMSD and radius of gyration (Rg) as collective variables on equilibrated trajectories to profile the global conformational free energy distribution of the complexes. Representative conformations extracted from the global free energy minima were used to evaluate the binding stability of the systems.

### 2.3 SMD and US simulations

To thoroughly investigate the binding stability between the selected hit compounds and ALK, enhanced sampling MD simulations were performed using the GROMACS 2023.1 (single-precision version) software package. The dissociation free energy calculation was accomplished via a two-step method combining SMD and US.

During the SMD simulation stage, the pull module of GROMACS was utilized to drive the hit compounds along the positive Z-axis direction for acquiring continuous conformations along the dissociation pathway. A harmonic restraining force with a spring constant of 3,000 kJ/(mol⋅nm^2^) was applied at the ligand centroid, and the pulling speed was set to 0.02 nm/ps. Conformations of the ALK–hit compound complex were recorded every 0.2 nm of displacement, and these conformations were employed as the initial conformations for subsequent US simulations to ensure complete coverage of the pathway from the bound state to the fully dissociated state.

For US simulations, the aforementioned continuous conformations were selected for segmental sampling. Each initial conformation underwent an NPT ensemble equilibration simulation with a duration of 0.1 ns. After the system achieved thermodynamic stability, a 10 ns US simulation was conducted. All US simulation results were integrated using the Weighted Histogram Analysis Method (WHAM), with the specific parameters set as follows: the histogram bin size was 0.025 nm; the global energy convergence tolerance for WHAM was fixed at 10^−6^ kJ/mol; the convergence criterion for the Potential of Mean Force (PMF) curve was defined as follows: when the pulling distance (ξ) entered the displacement interval corresponding to the dissociated state, if the fluctuation amplitude of the PMF values of no less than five consecutive displacement points relative to the average value of their respective interval was less than 5%, the PMF curve was deemed to have converged, and this interval was designated as the plateau region of the dissociated state. The dissociation free energy (ΔG) was defined as the difference between the average free energy of the ligand in the fully dissociated state after PMF curve convergence and that in the stable bound state (the free energy corresponds to the minimum value of the PMF curve).

To evaluate the statistical uncertainty of PMF estimation, 100 bootstrap resampling analyses were performed by invoking the -nBootstrap parameter using the gmx wham tool integrated in GROMACS. Subset data with replacement were randomly extracted from the original umbrella sampling trajectories, and the WHAM fitting and PMF calculation processes were repeated. Finally, the bootstrap standard error of each data point in the PMF curve was obtained (presented as error bars in the PMF curve).

### 2.4 Cell culture

Four human cell lines, including three NB cell lines (SK-N-BE (2), SH-SY5Y, IMR-32) and the ALK-negative human embryonic kidney cell line HEK293T, were purchased from Procell Life Science & Technology Co., Ltd. (Wuhan, China). All cell lines were authenticated by the supplier via short tandem repeat (STR) profiling to confirm cell identity and absence of cross-contamination. Each cell line was maintained in cell-specific culture medium (all media from Procell, China): SK-N-BE (2) was cultured in DMEM/F12; IMR-32 was maintained in MEM supplemented with non-essential amino acids (NEAA, Procell, China); both SH-SY5Y and HEK293T cells were cultured in high-glucose DMEM. All media were supplemented with 10% fetal bovine serum (FBS, Procell, China) and 1% antimicrobial solution (containing 100 U/mL penicillin, 100 μg/mL streptomycin, and 50 μg/mL gentamicin, sevenbio, China) to prevent microbial contamination. Cells were cultured at 37 °C in a humidified atmosphere with 5% CO_2_, with fresh medium replaced every 2–3 days. For passaging, cells were harvested using 0.25% trypsin-EDTA (sevenbio, China) when reaching optimal confluency: NB cell lines were passaged at 80%–90% confluency, while HEK293T cells were passaged at 70%–80% confluency. The detailed passaging procedure was consistent across all cell lines: culture medium was aspirated, cells were rinsed twice with PBS, and trypsin-EDTA was added for 2–3 min to induce cell detachment, which was immediately terminated by adding fresh complete medium. The cell suspension was centrifuged at 1,000 × g for 5 min; the supernatant was discarded, and the cell pellet was resuspended and reseeded into new flasks at an appropriate split ratio. All cells were maintained in the logarithmic growth phase to provide a stable cell source for subsequent experiments.

### 2.5 Cell viability assay

Cell viability was measured using the Cell Counting Kit-8 (CCK-8) assay across all tested cell lines, including three NB cell lines (SK-N-BE (2), IMR-32, SH-SY5Y) and HEK293T cells, to evaluate the antiproliferative effects of test compounds. Briefly, cells in the logarithmic growth phase were harvested and resuspended in their corresponding complete culture medium supplemented with 10% FBS. The cell density was adjusted to 5 × 10^3^ cells per well, and cell suspensions were seeded into 96-well plates at 150 μL per well, with three technical replicate wells set for each treatment group. After 24 h of incubation to allow complete cell adherence, the original culture medium was aspirated. For NB cell lines, fresh medium containing hit compounds or the positive control AZD-3463 at serial concentrations (0, 0.01, 0.1, 1, 10, 20 μM) was added to each well, with a final volume of 100 μL per well. For HEK293T cells, fresh medium containing gradient concentrations of C18 (0, 0.01, 0.1, 1, 10, 20 μM) was added following the same volume setting. For all cell lines, the final concentration of dimethyl sulfoxide (DMSO) in each well was strictly controlled to ≤0.1%, and vehicle control wells received an equal volume of medium containing the same final concentration of DMSO. After drug administration, plates were incubated at 37 °C with 5% CO_2_ for another 24 h. Subsequently, 10 μL of CCK-8 reagent was added to each well, followed by 2 h of further incubation. The absorbance (optical density, OD value) of each well at 450 nm was measured using a microplate reader. Cell viability was calculated according to the formula: Cell viability (%) = (OD value of experimental group/ OD value of vehicle control group) × 100%. For NB cell lines, dose-response curves were fitted using GraphPad Prism 10 software to calculate the half-maximal inhibitory concentration (IC_50_) and 95% confidence interval (95% CI) for each compound. All hit compounds and the positive control were purchased from MedChemExpress (New Jersey, USA), with detailed compound information provided in Supplementary Table S1. All CCK-8 assays were independently repeated three times to ensure result reliability.

### 2.6 Colony formation assay

Target cells in the logarithmically growing phase were harvested, digested with trypsin, and resuspended in culture medium supplemented with 10% fetal bovine serum (FBS). The cells were seeded into 6-well plates at a density of 500 cells per well, with replicate wells set for each group. After seeding, the plates were incubated at 37 °C with 5% CO_2_. For adherent cells, drug treatment was administered after 24 h of incubation; fresh medium was replaced every 3 days, and the culture was continued for 1–2 weeks. The medium was discarded, and the wells were gently rinsed with PBS, followed by fixation with 4% paraformaldehyde for 15–20 min and staining with 0.5% crystal violet solution for 10–15 min. After rinsing thoroughly with deionized water and air-drying, colonies containing ≥50 cells were counted.

### 2.7 EdU cell proliferation assay

EdU detection was performed per the instructions of BeyoClick EdU-594 Kit (C0078, Beyotime, China): A 2×EdU working solution was prepared, added to each well to reach the experimental final concentration, and incubated at 37 °C for 2 h. The solution was discarded; 4% paraformaldehyde was added for fixation at RT for 15 min, followed by 3 PBS washes. PBS with 0.3% Triton X-100 was then added for permeabilization at RT for 10–15 min, and wells were washed 1–2 times with PBS. Click reaction solution was prepared (per 500 μL: 430 μL reaction buffer, 1 μL Azide 594, 20 μL CuSO_4_, 49 μL Click additive in deionized water), added to each well, and incubated at RT for 30 min in the dark. The reaction solution was aspirated, and wells were washed 3 times with PBS. DAPI staining solution was added for nuclear staining at RT for 10 min in the dark; after PBS washing, images were captured via inverted fluorescence microscope. EdU-positive and total cells were counted using ImageJ, and the EdU-positive rate was calculated.

### 2.8 Transwell assay

Logarithmically growing SK-N-BE (2) and IMR-32 cells were resuspended in respective serum-free media and adjusted to 2 × 10^5^ cells/mL. 600 μL of corresponding medium containing 20% FBS (as chemoattractant) was added to the lower chamber of 24-well plates, followed by Transwell inserts (8 μm pore size, BIOFIL, China). 200 μL of cell suspension with corresponding drugs was added to the upper chambers, and incubated at 37 °C with 5% CO_2_ for 48 h. After incubation, inserts were removed; medium in upper chambers was aspirated, and the upper surfaces of insert membranes were washed twice with PBS to wipe off non-migrated cells. Inserts were immersed with membrane downward in 4% paraformaldehyde for fixation at room temperature (RT) for 30 min, rinsed thoroughly with PBS, then stained with 0.1% crystal violet solution for 20 min at RT. Excess stain was washed off with running water, and inserts were air-dried upside down. Images were captured under an inverted microscope, and migrated cells in each group were counted using ImageJ software. The experiment was independently repeated at least three times.

### 2.9 Western blot analysis

The treated cells were lysed on ice bath with RIPA lysis buffer (Beyotime, China) containing 1% Protease Inhibitor Cocktail (TargetMol, United States) and 1% Phosphatase Inhibitor Cocktail I (TargetMol, USA). After determining the protein concentration using a BCA protein quantification kit (Beyotime, China), the protein samples were subjected to SDS-PAGE for separation, then electrotransferred onto PVDF membranes, followed by blocking of non-specific binding sites on the membrane surface with TBST buffer containing 5% skimmed milk. The blocked membranes were incubated with primary antibodies at 4 °C overnight, washed three times (10 min each) with TBST buffer, then incubated with HRP-conjugated IgG (H + L) secondary antibody (Beyotime, China) diluted at 1:1000 for 1 h at RT, and washed three times again using the same method. Finally, the antigen-antibody complexes were detected with ECL chemiluminescent reagent (Beyotime, China), and images were collected by exposure with a gel imaging system. Using GAPDH as the internal reference, the gray values of the bands were analyzed by ImageJ software. The antibodies used in the experiment included: ALK (Cat. No.: A01468-2, Boster Biological Technology Co., Ltd., China), BCL-2 (Cat. No.: A00040-1, Boster Biological Technology Co., Ltd., China), PARP (Cat. No.: PB9309, Boster Biological Technology Co., Ltd., China), Cleaved PARP-N (Cat. No.: BM3941, Boster Biological Technology Co., Ltd., China), GAPDH (Cat. No.: A00227-1, Boster Biological Technology Co., Ltd., China), AKT (Cat. No.: 10176-2-AP, Proteintech Group, Inc., China), phosphorylated AKT (P-AKT, Cat. No.: 66444-1-Ig, Proteintech Group, Inc., China), mTOR (Cat. No.: 66888-1-Ig, Proteintech Group, Inc., China), phosphorylated mTOR (P-mTOR, Cat. No.: 80596-1-RR, Proteintech Group, Inc., China).

### 2.10 Quantitative real-time PCR (qPCR)

Total RNA was extracted from the treated cells using a spin column-based total RNA extraction kit (Seven Innovation, Beijing, China). Reverse transcription and quantitative real-time PCR were performed using the SevenFast® Two Step RT&qPCR Kit (Seven Innovation, Beijing, China). Briefly, 1 μg of total RNA was reverse transcribed into cDNA according to the manufacturer’s instructions (42 °C for 15 min, followed by 95 °C for 3 min). The qPCR reaction was carried out using 2× SYBR Green qPCR Mix with specific primers and cDNA template on a real-time PCR system under the following conditions: 95 °C for 30 s, followed by 40 cycles of 95 °C for 5 s and 60 °C for 30 s. The primer sequences were as follows: GAPDH forward GGA​GCG​AGA​TCC​CTC​CAA​AAT, reverse GGC​TGT​TGT​CAT​ACT​TCT​CAT​GG; ALK forward TCT​CAT​CGC​AGC​CGA​TAT​GG, reverse GGC​ATC​TCC​TTA​GAA​CGC​TCT. Relative ALK mRNA expression was calculated using the 2^−ΔΔCt method with GAPDH as the internal reference.

### 2.11 Immunofluorescence (IF) assay

Treated cells in 24-well plates were washed with pre-chilled 1×PBS after medium removal, fixed with 4% paraformaldehyde, and washed with 1×PBST (0.05% Tween-20). Following permeabilization with 1×PBS +0.3% Triton X-100 and PBST washing, cells were blocked with 1×PBS +5% BSA (blocking buffer discarded without washing). ALK primary antibody (1:100 in 1×PBS +1% BSA) was incubated overnight at 4 °C in the dark; after PBST washing, 488-conjugated goat anti-rabbit IgG (H + L) (1:200 in 1×PBS +1% BSA) was incubated for 1 h at RT in the dark. Cells were washed with PBST and 1×PBS, stained with DAPI (1–5 μg/mL) in the dark, washed again with 1×PBS, mounted with anti-fade medium (no bubbles), and imaged via fluorescence microscope. Experiments were performed in three independent replicates.

### 2.12 Flow cytometry analysis of apoptosis

Cell apoptosis was detected using the Annexin V-FITC/PI Apoptosis Detection Kit (Sevenbio, China). Target cells in the logarithmically growing phase were harvested and seeded into 6-well plates at a density of 2 × 10^5^ cells per well, followed by incubation for 24 h to allow complete adherence. After 24 h of drug treatment, floating cells in the supernatant were collected first; adherent cells were digested with trypsin without EDTA (to avoid interfering with Ca^2+^-dependent Annexin V binding), and the two cell suspensions were combined. The cells were centrifuged at 1,000 × g for 5 min, washed twice with pre-cooled PBS, and resuspended in 100 μL of Annexin V binding buffer. After adding 5 μL of Annexin V-FITC and 5 μL of PI staining solution, the mixture was incubated at RT in the dark for 15 min. Subsequently, 400 μL of binding buffer was supplemented, and detection was performed on a flow cytometer within 1 h. Cells stained as Annexin V^+^/PI^−^ were defined as early apoptotic cells, Annexin V^+^/PI^+^ as late apoptotic cells, and the total apoptosis rate was calculated as the sum of these two populations.

### 2.13 Drug affinity responsive target stability (DARTS) assay

Referring to previous methods (Yin et al., 2025), NB cells were cultured to full confluency, harvested by mechanical scraping, and transferred to 1.5 mL centrifuge tubes. The cells were centrifuged at 4 °C and 1,000 × g for 5 min; the supernatant was discarded, and the cell pellet was maintained on ice. Subsequently, 100 μL of ice-cold lysis buffer was added for cell lysis, followed by incubation on ice for 10 min. After centrifugation at 4 °C and 14,000 × g for 20 min, the supernatant was collected. The protein concentration of the supernatant was determined via the BCA assay and adjusted to 10 μg/μL with ice-cold PBS. A 40 μL aliquot of the protein sample was equally divided into four groups, with corresponding reagents added: Group A (DMSO control), Group B (DMSO + Pronase E), Group C (CDK12-IN-3 + Pronase E), and Group D (AZD-3463 + Pronase E, positive control). After all groups were incubated overnight at 4 °C, TNC buffer was added to each group, and an additional 2 μL of 2.5 mg/mL Pronase E (Cat. No.: HY-114158, MCE, USA) was added to Groups B–D. Following 15 min of proteolysis at RT, the samples were analyzed by Western blot.

### 2.14 Surface plasmon resonance (SPR) analysis

The binding affinity between ALK protein and CDK12-IN-3 was measured using a Biacore 1K instrument (Cytiva, Marlborough, MA, United States). Recombinant ALK protein (50 μg/mL in 10 mM sodium acetate buffer, pH 4.5) was covalently immobilized on a CM5 sensor chip (Cytiva) via standard amine coupling chemistry using EDC/NHS activation, followed by blocking with 1 M ethanolamine-HCl (pH 8.5). A reference flow cell was activated and blocked without protein immobilization for background subtraction. All interaction experiments were performed at 25 °C in running buffer (1× PBS-P+, pH 7.4, supplemented with 5% DMSO). CDK12-IN-3 was serially diluted two-fold in running buffer to concentrations ranging from 0.3125 to 10 μM, and injected over the sensor chip surface at a flow rate of 30 μL/min for an association phase of 60 s, followed by a dissociation phase. The sensor chip surface was regenerated with 10 mM glycine-HCl (pH 2.0) between each concentration cycle. Solvent correction was applied to compensate for DMSO bulk refractive index effects. Sensorgrams were double-referenced by subtracting the reference flow cell response and buffer blank response, and kinetic parameters were determined by global fitting to a 1:1 Langmuir binding model using Biacore Insight Evaluation Software (Cytiva, version 6.0).

### 2.15 *In vivo* experimental animals and tumor xenograft model establishment and treatment

Twelve 4-week-old Balb/c nude mice were used as experimental animals in this study. All mice were housed under SPF conditions with a 1 week acclimatization period before experiments. Throughout the housing period and subsequent experiments, the experimental animal welfare and ethical standards as well as SPF environmental management regulations were strictly followed. Subsequently, on day −7 of the experiment (7 days before the initiation of administration), each nude mouse was inoculated with a suspension of pretreated murine NB cell line (Neuro-2a) via unilateral subcutaneous injection at a density of 1 × 10^6^ cells per mouse to establish a subcutaneous xenograft tumor model. On day 0 of the experiment, after confirmation of successful tumor formation in all mice by palpation combined with morphological observation, the tumor volumes were measured. The mice were then randomly divided into two groups (n = 6 per group) using the normal distribution and equidistant grouping method: the vehicle control group (Vehicle group) and the CDK12-IN-3 administration group, ensuring consistent baseline tumor volumes between the two groups. From day 0 onwards, gavage administration was performed once daily: mice in the CDK12-IN-3 group received CDK12-IN-3 at a dose of 50 mg/kg, while those in the Vehicle group received an equal volume of vehicle. The administration was continued until the experimental endpoint. During the treatment period, the maximum length (L) and perpendicular width (W) of the tumors were measured daily using high-precision vernier calipers with a resolution of 0.01 mm, and the tumor volume was calculated and recorded using formula V = L × W^2^/ 2. At the experimental endpoint, tumor-bearing mice were anesthetized with isoflurane, and their general appearance was photographed to record the physiological status. Subsequently, euthanasia was performed via cervical dislocation. The tumor xenografts were completely dissected, and their macroscopic morphological characteristics were recorded. Each tumor tissue was cut longitudinally into two parts: one part was fixed with 4% paraformaldehyde for IHC assay, and the other part was snap-frozen in liquid nitrogen for subsequent WB assay.

### 2.16 Immunohistochemistry

4% paraformaldehyde-fixed, paraffin-embedded tissues were cut into serial 3–5 μm sections, mounted on poly-L-lysine slides, and baked at 60 °C for 2 h. Sections were dewaxed in xylene I/II (10 min each), hydrated in graded ethanol (100%–75%, 5 min/concentration), and rinsed 3× with PBS (pH 7.4, 5 min each). Antigen retrieval: heated in boiling 0.01 M citrate buffer (pH 6.0) at gentle boil for 10 min, cooled to RT, and rinsed 3× with PBS. Endogenous peroxidase blocked with 3% H_2_O_2_ (15 min, RT, dark); non-specific binding blocked with 5% BSA (30 min, RT). Primary antibody (manufacturer-recommended dilution) incubated overnight at 4 °C (humidified chamber); after rewarming and PBS rinses, HRP-conjugated secondary antibody (species-matched, per instructions) incubated for 30 min at RT. PBS-rinsed sections stained with fresh DAB (3–5 min, microscopically monitored), reaction terminated with tap water. Hematoxylin counterstaining (5 min), differentiated in 1% HCl-ethanol, rinsed, and blued in 1% ammonia water (2 min). Finally, dehydrated in graded ethanol (75%–100%, 3 min each), cleared in xylene I/II (5 min each), mounted in neutral balsam, and air-dried at RT.

### 2.17 Statistical analysis

The experimental results were statistically analyzed using GraphPad Prism 10.0 software. All experiments were performed with at least three independent biological replicates (n = 3), and data are expressed as mean ± SEM. Comparisons between two groups were performed using the two-tailed Student’s t-test. A P value <0.05 was considered statistically significant.

## 3 Results

**FIGURE 1:**
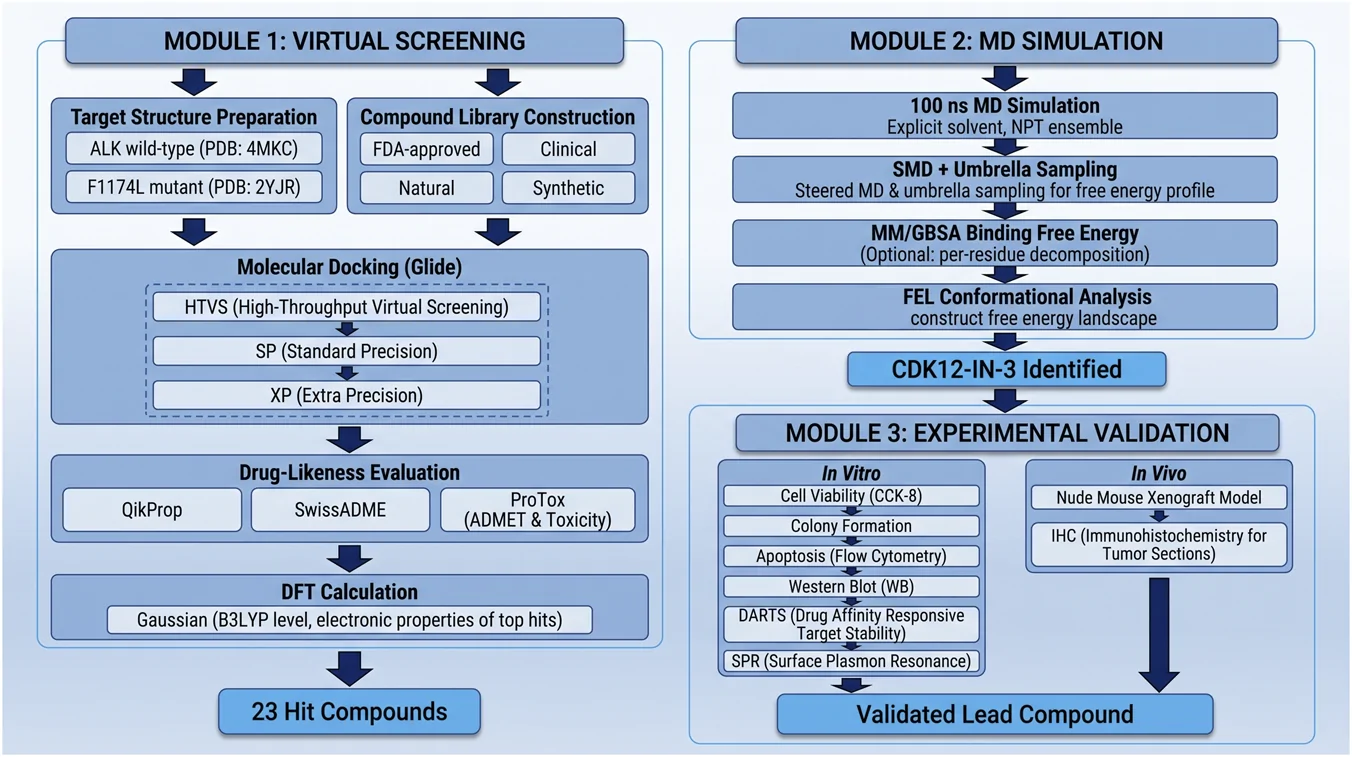
Schematic overview of the integrated workflow for the identification and validation of CDK12-IN-3 as a potent ALK inhibitor.

The overall research workflow of this study is illustrated in Figure 1, which integrates virtual screening, multi-dimensional molecular dynamics simulations, and *in vitro*/*in vivo* experimental validation into a cohesive drug repurposing framework.

### 3.1 Virtual screening for potential ALK-Inhibiting hit compounds

After LigPrep preprocessing and sequential HTVS, SP, and XP docking (top 30% retained per round), 23 hit compounds with potential binding activity to both wild-type ALK and ALK(F1174L) were identified; their basic information is presented in Table 1. The druggability and safety profiles of the 23 screened hit compounds were evaluated via *in silico* prediction using the SwissADME and Protox 3.0 web servers (Supplementary Tables S2, S3). The results identified potential druggability and safety risks in a subset of hits: for structural alerts, C1–C3 carried quinone-type PAINS alerts, C4 harbored dual PAINS alerts (quinone and anthranilamide), and C6 exhibited catechol-type PAINS alerts; for toxicity risks, seven compounds (C1, C2, C3, C4, C9, C11, C17) were predicted to carry mutagenic potential, while C7 and C9 showed predicted hepatotoxicity risks.

**TABLE 1:** Basic information of hit compounds.

S.No	Compound	MF	Structural formula	S.No	Compound	MF	Structural Formula
C1	Emodin	C_15_H_10_O_5_	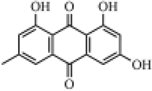	C10	PHA-793887	C_19_H_31_N_5_O_2_	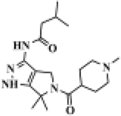
C2	Chrysophanol	C_15_H_10_O_4_	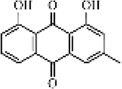	C11	BS-181 hydrochloride	C_22_H_33_ClN_6_	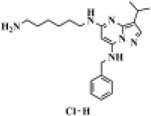
C3	Physcion	C_16_H_12_O_5_	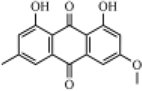	C12	AZ3146	C_24_H_32_N_6_O_3_	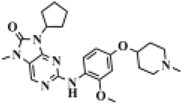
C4	Mitoxantrone	C_22_H_28_N_4_O_6_	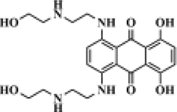	C13	Onatasertib	C_21_H_27_N_5_O_3_	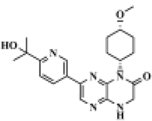
C5	(R)-Roscovitine	C_19_H_26_N_6_O	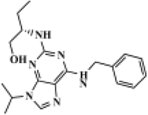	C14	CC-401	C_22_H_24_N_6_O	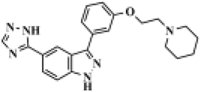
C6	Fisetin	C_15_H_10_O_6_	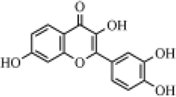	C15	UNC2025	C_28_H_40_N_6_O	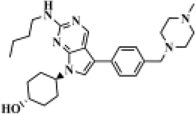
C7	Morin	C_15_H_10_O_7_	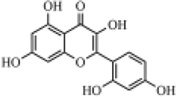	C16	KW-2449	C_20_H_20_N_4_O	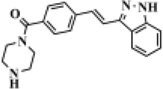
C8	Cvt-313	C_20_H_28_N_6_O_3_	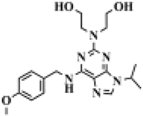	C17	Dinaciclib	C_21_H_28_N_6_O_2_	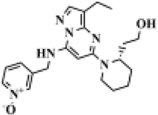
C9	APY29	C_17_H_16_N_8_	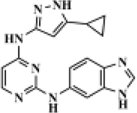	C18	CDK12-IN-3	C_23_H_28_F_2_N_8_O	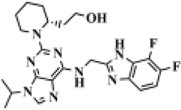
C19	Voruciclib	C_22_H_19_ClF_3_NO_5_	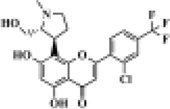	C22	Purvalanol A	C_19_H_25_ClN_6_O	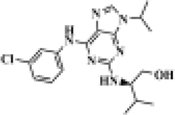
C20	LDC4297	C_23_H_28_N_8_O	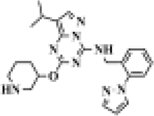	C23	Ca^2+^ channel agonist 1	C_19_H_26_N_6_O	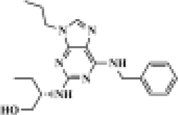
C21	LX7101	C_23_H_29_N_7_O_3_	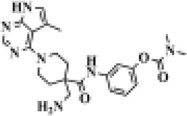	​	​	​	​

DFT calculations were performed on the 24 candidate compounds (C1–C24) to evaluate their geometric stability and electronic properties prior to MD simulations (Supplementary Table S4). Geometry optimization confirmed the absence of imaginary frequencies across all compounds, indicating that all optimized structures correspond to thermodynamically stable ground-state conformations. The dipole moments ranged from 1.38 D (C5) to 18.40 D (C19). Given that ALK is a cytoplasmic kinase, compounds with substantially elevated dipole moments may exhibit compromised passive membrane permeability, as highly polar molecules experience an increased free energy barrier when crossing the hydrophobic membrane core (Bernardi et al., 2023). Notably, C16 (9.46 D), C19 (18.40 D), and C21 (8.34 D) displayed dipole moments exceeding 8 D, suggesting potentially poor cell penetration that warrants attention in subsequent development. The HOMO–LUMO energy gap (Egap) varied considerably across the compound set (0.32–4.82 eV). C18 exhibited an Egap of 3.90 eV, falling within the range typically associated with kinetically stable, drug-like molecules. Collectively, these DFT-derived parameters provided complementary physicochemical insights that, together with the virtual screening results, supported the prioritization of candidates for subsequent MD-based binding assessment.

### 3.2 MD simulation and US simulation for screening high-affinity ligands of ALK from hit compounds

To further quantitatively analyze the binding affinity between candidate compounds and ALK, this study prioritized MD simulations of complexes formed by wild-type ALK, two key mutant ALK variants (F1174L, R1275Q), and the candidate compounds. Trajectory data from the equilibrium phase (50–100 ns) of all simulations were selected, and the binding free energy (ΔGbind) of each complex was calculated using the MM/PBSA method. As shown in Table 2, the binding capacities of compounds to wild-type ALK and two major ALK mutants (F1174L, R1275Q) in NB were ranked by ΔGbind values in ascending order (i.e., binding affinity in descending order). The results revealed that compounds C4, C15, C18, and C20 exhibited top-10 level high affinity to both wild-type ALK and the major NB-associated ALK mutants (F1174L, R1275Q), making them potential high-affinity ALK ligands.

**TABLE 2:** MM/PBSA binding free energies of hit compounds and positive control with ALK wild-type and mutant types (kcal/mol).

Complex	ΔGbind	Complex	ΔGbind	Complex	ΔGbind
ALK(Wild-type) - C1	−31.40 ± 0.81	ALK(F1174L) - C1	−35.01 ± 2.08	ALK (R1275Q) - C1	−31.49 ± 2.68
ALK(Wild-type) - C2	−26.94 ± 0.73	ALK(F1174L) - C2	−29.42 ± 2.38	ALK (R1275Q) - C2	−27.76 ± 0.62
ALK(Wild-type) - C3	−27.39 ± 0.94	ALK(F1174L) - C3	−28.50 ± 1.77	ALK (R1275Q) - C3	−25.71 ± 0.83
ALK(Wild-type) - C4	−41.33 ± 3.33	ALK(F1174L) - C4	−40.70 ± 4.55	ALK (R1275Q) - C4	−55.13 ± 6.49
ALK(Wild-type) - C5	−38.14 ± 1.11	ALK(F1174L) - C5	−38.33 ± 1.60	ALK (R1275Q) - C5	−36.73 ± 0.66
ALK(Wild-type) - C6	−35.19 ± 2.15	ALK(F1174L) - C6	−42.51 ± 4.04	ALK (R1275Q) - C6	−32.39 ± 4.79
ALK(Wild-type) - C7	−31.72 ± 2.35	ALK(F1174L) - C7	−29.72 ± 4.79	ALK (R1275Q) - C7	−32.75 ± 3.56
ALK(Wild-type) - C8	−39.55 ± 1.20	ALK(F1174L) - C8	−39.63 ± 0.83	ALK (R1275Q) - C8	−41.94 ± 1.16
ALK(Wild-type) - C9	−41.80 ± 3.41	ALK(F1174L) - C9	−38.67 ± 1.98	ALK (R1275Q) - C9	−43.21 ± 3.38
ALK(Wild-type) - C10	−41.49 ± 2.04	ALK(F1174L) - C10	−40.19 ± 2.63	ALK (R1275Q) - C10	−42.06 ± 3.81
ALK(Wild-type) - C11	−35.51 ± 1.85	ALK(F1174L) - C11	−41.36 ± 6.93	ALK (R1275Q) - C11	−44.21 ± 4.17
ALK(Wild-type) - C12	−41.79 ± 1.05	ALK(F1174L) - C12	−39.54 ± 1.55	ALK (R1275Q) - C12	−39.53 ± 1.48
ALK(Wild-type) - C13	−29.55 ± 4.81	ALK(F1174L) - C13	−27.93 ± 5.01	ALK (R1275Q) - C13	−30.98 ± 3.31
ALK(Wild-type) - C14	−38.10 ± 0.91	ALK(F1174L) - C14	−34.14 ± 5.85	ALK (R1275Q) - C14	−42.28 ± 2.84
ALK(Wild-type) - C15	−48.22 ± 1.00	ALK(F1174L) - C15	−51.57 ± 4.14	ALK (R1275Q) - C15	−52.25 ± 2.37
ALK(Wild-type) - C16	−36.95 ± 2.95	ALK(F1174L) - C16	−34.60 ± 1.70	ALK (R1275Q) - C16	−43.65 ± 3.64
ALK(Wild-type) - C17	−42.70 ± 0.72	ALK(F1174L) - C17	−45.07 ± 1.84	ALK (R1275Q) - C17	−42.05 ± 1.32
ALK(Wild-type) - C18	−41.32 ± 1.70	ALK(F1174L) - C18	−40.64 ± 4.38	ALK (R1275Q) - C18	−42.75 ± 0.84
ALK(Wild-type) - C19	−44.71 ± 2.17	ALK(F1174L) - C19	−44.21 ± 4.20	ALK (R1275Q) - C19	445.51 ± 2.98
ALK(Wild-type) - C20	−41.57 ± 1.42	ALK(F1174L) - C20	−40.98 ± 3.01	ALK (R1275Q) - C20	−47.11 ± 2.29
ALK(Wild-type) - C21	−41.56 ± 2.20	ALK(F1174L) - C21	−36.50 ± 6.96	ALK (R1275Q) - C21	−42.09 ± 2.53
ALK(Wild-type) - C22	−39.85 ± 1.41	ALK(F1174L) - C22	−39.21 ± 1.57	ALK (R1275Q) - C22	−35.92 ± 1.25
ALK(Wild-type) - C23	−37.96 ± 0.62	ALK(F1174L) - C23	−38.38 ± 0.74	ALK (R1275Q) - C23	−43.06 ± 2.80
ALK(Wild-type) - AZD-3463	−39.24 ± 1.61	ALK(F1174L) - AZD-3463	−42.42 ± 3.35	ALK (R1275Q) - AZD-3463	−44.16 ± 1.31

To further evaluate the binding robustness between four high-affinity ALK ligands (C4, C15, C18, C20) screened via MM/PBSA calculation, positive control C24 (AZD-3463), and ALK, this study first analyzed the backbone RMSD of ALK in complexes formed by wild-type ALK, mutant ALK (F1174L), ALK (R1275Q) with each ligand, respectively, during 100 ns MD simulations (including five sets of replicate experiments with random initial velocities).

Supplementary Figure S1 shows the changes in backbone RMSD of the three ALK subtypes (after binding different ligands) in five sets of random simulations with curves of different colors. The results revealed that the backbone RMSD of ALK (wild-type and two mutants) in all complexes converged rapidly at the early stage of simulations and did not exhibit continuous large fluctuations throughout the process.

In this study, the root mean square deviation (ligand_RMSD) of ligand non-hydrogen atoms relative to the backbones of three ALK subtypes (wild-type, ALK (F1174L), and ALK (R1275Q)) was further analyzed to quantify the spatial fluctuation degree and conformational stability of ligands relative to ALK in the binding pocket.

**FIGURE 2:**
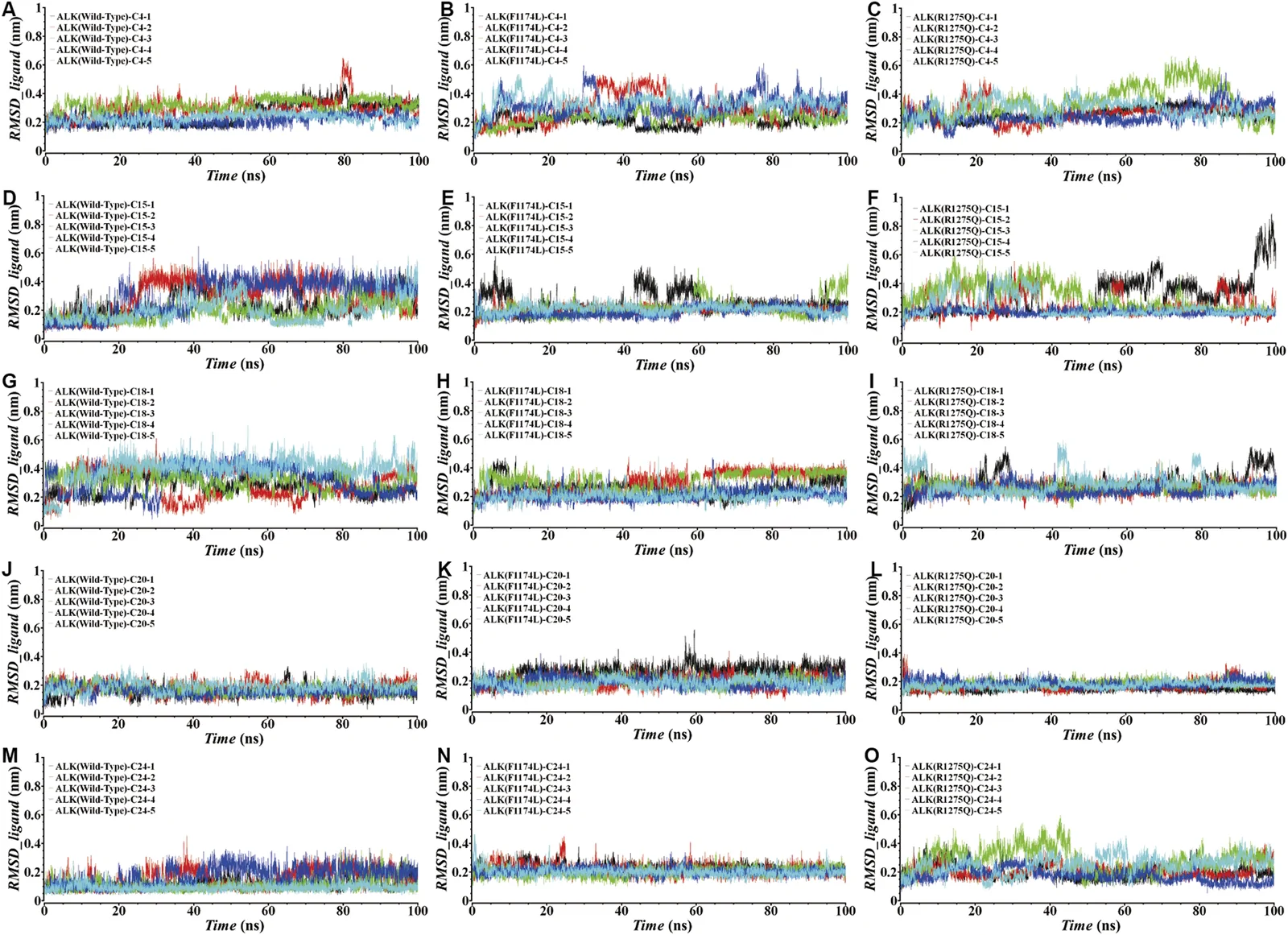
Ligand RMSD profiles of ALK-hit compound complexes and positive control over 100 ns MD simulation. **(A)** ALK(WT)-C4. **(B)** ALK(F1174L)-C4. **(C)** ALK(R1275Q)-C4. **(D)** ALK(WT)-C15. **(E)** ALK(F1174L)-C15. **(F)** ALK(R1275Q)-C15. **(G)** ALK(WT)-C18. **(H)** ALK(F1174L)-C18. **(I)** ALK(R1275Q)-C18. **(J)** ALK(WT)-C20. **(K)** ALK(F1174L)-C20. **(L)** ALK(R1275Q)-C20. **(M)** ALK(WT)-C24. **(N)** ALK(F1174L)-C24. **(O)** ALK(R1275Q)-C24.

As shown in Figure 2, in five sets of random simulations, the ligand_RMSD curves of complexes formed by C4, C15, C18, C20, and the positive control C24 with most ALK subtypes exhibited good stability: the RMSD values of these complexes mostly remained in a low-fluctuation range of 0.2–0.4 nm, without obvious sudden surge peaks or continuous increases; additionally, the RMSD curves of replicate simulations in each group showed high coincidence and small fluctuation amplitude. However, the complexes formed by ALK (R1275Q) subtype with C4 and C15 were exceptions—the spatial fluctuation amplitude of ligands relative to ALK in these two complexes increased significantly: specifically, the RMSD value of ALK (R1275Q)-C4 deviated from the low-stability range and showed an obvious fluctuation peak in the third simulation; for ALK (R1275Q)-C15, the RMSD value surged sharply and peaked at nearly 1 nm in the first simulation, indicating extremely poor conformational stability of the ligand relative to ALK in these two simulations. Overall, C18, C20, and most other complexes still exhibited good binding stability and could be regarded as potential high-affinity ALK ligands.

FEL were further constructed using protein backbone RMSD and R_g_ as collective variables to characterize the global conformational stability of equilibrated ALK-ligand complexes (Supplementary Figures S2–S4). Only the C20-ALK(F1174L) mutant complex harbored two low-energy conformational substates with comparable free energy levels, while all other complexes formed by C4, C15, C18, and the positive control C24 with the three ALK subtypes presented a clear, well-defined single low-energy basin. Analysis of representative conformations extracted from the global free energy minimum of each system confirmed that all ligands remained stably anchored in the ATP-binding pocket of ALK, with key hydrogen bonds and hydrophobic interactions well preserved, indicating that all complexes maintained stable binding throughout the equilibrium simulations.

**FIGURE 3:**
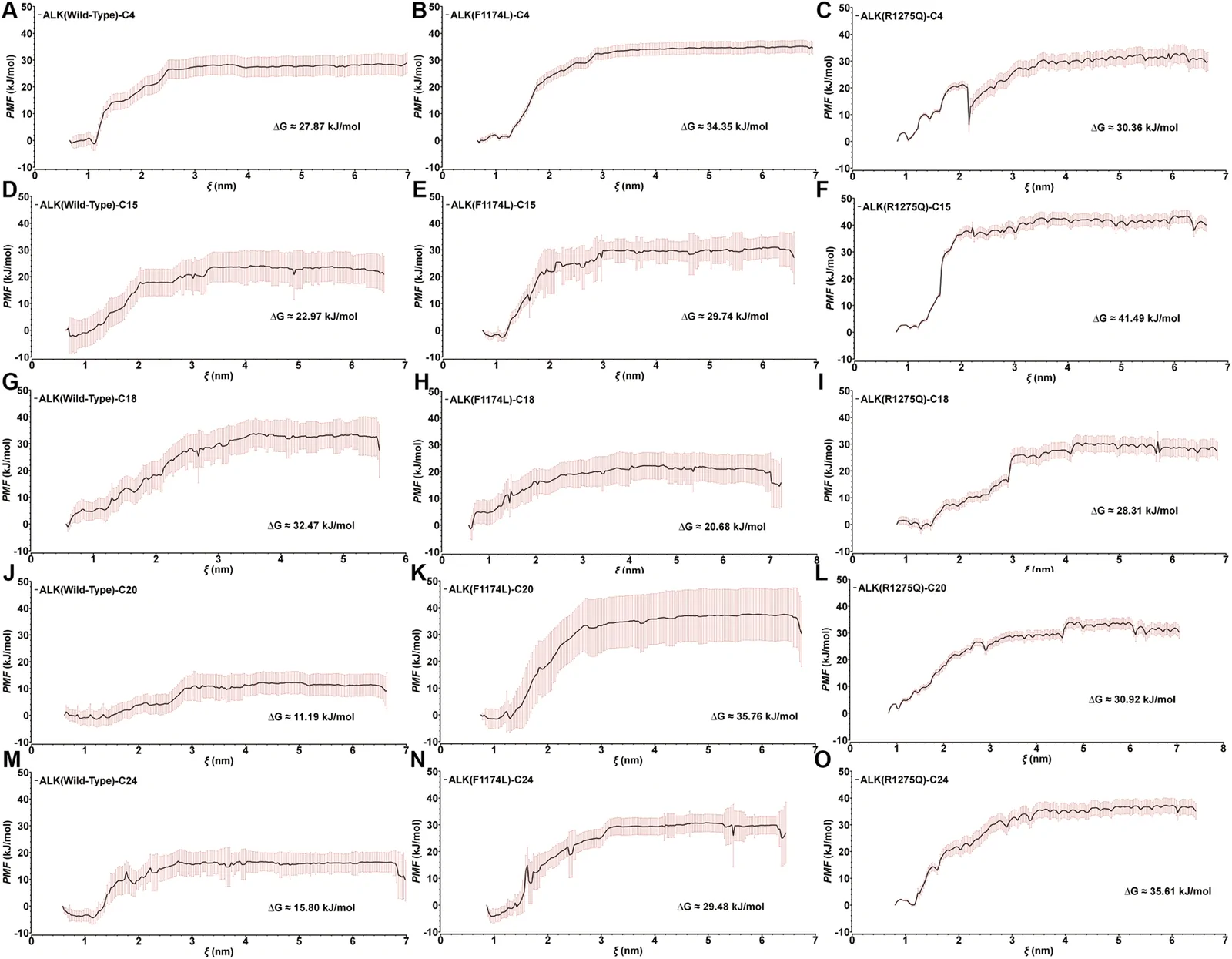
Dissociation free energy curves of complexes formed by wild-type ALK, ALK(F1174L), and ALK(R1275Q) with optimized compounds. **(A)** ALK(WT)-C4. **(B)** ALK(F1174L)-C4. **(C)** ALK(R1275Q)-C4. **(D)** ALK(WT)-C15. **(E)** ALK(F1174L)-C15. **(F)** ALK(R1275Q)-C15. **(G)** ALK(WT)-C18. **(H)** ALK(F1174L)-C18. **(I)** ALK(R1275Q)-C18. **(J)** ALK(WT)-C20. **(K)** ALK(F1174L)-C20. **(L)** ALK(R1275Q)-C20. **(M)** ALK(WT)-C24. **(N)** ALK(F1174L)-C24. **(O)** ALK(R1275Q)-C24.

SMD-US simulations were used to calculate the dissociation energies of four candidate compounds and the positive control C24 in complex with wild-type ALK and NB-associated mutants (F1174L, R1275Q). The PMF profiles of all systems exhibited favorable convergence, and 100 bootstrap resampling trials revealed minor computational errors, verifying the reliability of the obtained data. As shown in Figure 3, overall, the dissociation energies of complexes formed by the four optimized compounds with various ALK systems were comparable to or higher than those of the positive control C24. The dissociation energies of most systems fell within the range of 27–42 kJ/mol, indicating that most candidate compounds formed highly stable complexes with ALK featuring high dissociation barriers and prolonged residence time. Only a few systems exhibited suboptimal low dissociation energies prone to unbinding: the complex of compound C20 with wild-type ALK possessed a dissociation energy of merely 11.19 kJ/mol, corresponding to the easiest dissociation and shortest residence time; the complex of compound C18 with F1174L mutant showed a relatively low dissociation energy of 20.68 kJ/mol; the positive control C24 bound to wild-type ALK yielded a dissociation energy of 15.89 kJ/mol, reflecting insufficient residence capacity against wild-type ALK.

### 3.3 Computational assessment of target selectivity of C18 for ALK over homologous tyrosine kinases

To evaluate the binding selectivity of C18 for ALK over structurally related kinases, MM-PBSA binding free energy calculations were performed on C18 complexed with wild-type ALK, two prevalent NB-associated ALK mutants (F1174L and R1275Q), and a panel of ten homologous human tyrosine kinases (EGFR, RET, ROS1, MET, KIT, AXL, TRKC, TRKB, TRKA, and FGFR2). Each complex was subjected to six independent 100 ns MD simulations, and the ΔGbind values are reported as mean ± standard deviation (Supplementary Table S5).

As shown in Supplementary Table S5, C18 exhibited consistently high binding affinity across all three ALK forms, with ΔGbind values of −41.32 ± 1.70 kcal/mol (wild-type), −40.64 ± 4.38 kcal/mol (F1174L), and −42.75 ± 0.84 kcal/mol (R1275Q), respectively. In contrast, the ΔGbind values for the ten homologous kinases ranged from −35.46 ± 4.31 kcal/mol (KIT) to −39.56 ± 1.15 kcal/mol (TRKB), all of which were markedly less favorable than those observed for any ALK variant. The mean ΔGbind difference between ALK (wild-type) and the closest non-ALK kinase (TRKB) was approximately 1.76 kcal/mol, corresponding to a calculated ΔΔGbind of −1.76 kcal/mol in favor of ALK. More pronounced differences were observed for other kinases: C18 bound EGFR (−36.72 ± 3.48 kcal/mol), ROS1 (−36.93 ± 2.74 kcal/mol), and KIT (−35.46 ± 4.31 kcal/mol) with ΔGbind values approximately 4–6 kcal/mol less favorable than to ALK, indicating a substantial binding preference for the intended target.

Among the non-ALK kinases examined, TRKB (−39.56 ± 1.15 kcal/mol), MET (−39.38 ± 1.02 kcal/mol), and FGFR2 (−39.07 ± 2.27 kcal/mol) displayed the most favorable ΔGbind values, yet all remained at least 1.76 kcal/mol weaker than C18’s binding to wild-type ALK. Notably, the binding affinity of C18 for the R1275Q mutant (−42.75 ± 0.84 kcal/mol) was the most favorable among all targets tested, with the smallest standard deviation, suggesting particularly stable recognition of this clinically relevant mutant.

Collectively, these computational data demonstrate that C18 exhibits preferential binding affinity for ALK (including clinically relevant mutant isoforms) over a broad panel of homologous tyrosine kinases. The ΔΔGbind differential of ≥1.76 kcal/mol between ALK and the closest non-ALK kinase, and of ≥4–6 kcal/mol for the majority of kinases tested, provides quantitative computational evidence supporting the target selectivity of C18 for ALK at the molecular level.

### 3.4 CCK-8 assay for screening ligands with high Anti-NB activity from hit compounds

**FIGURE 4:**
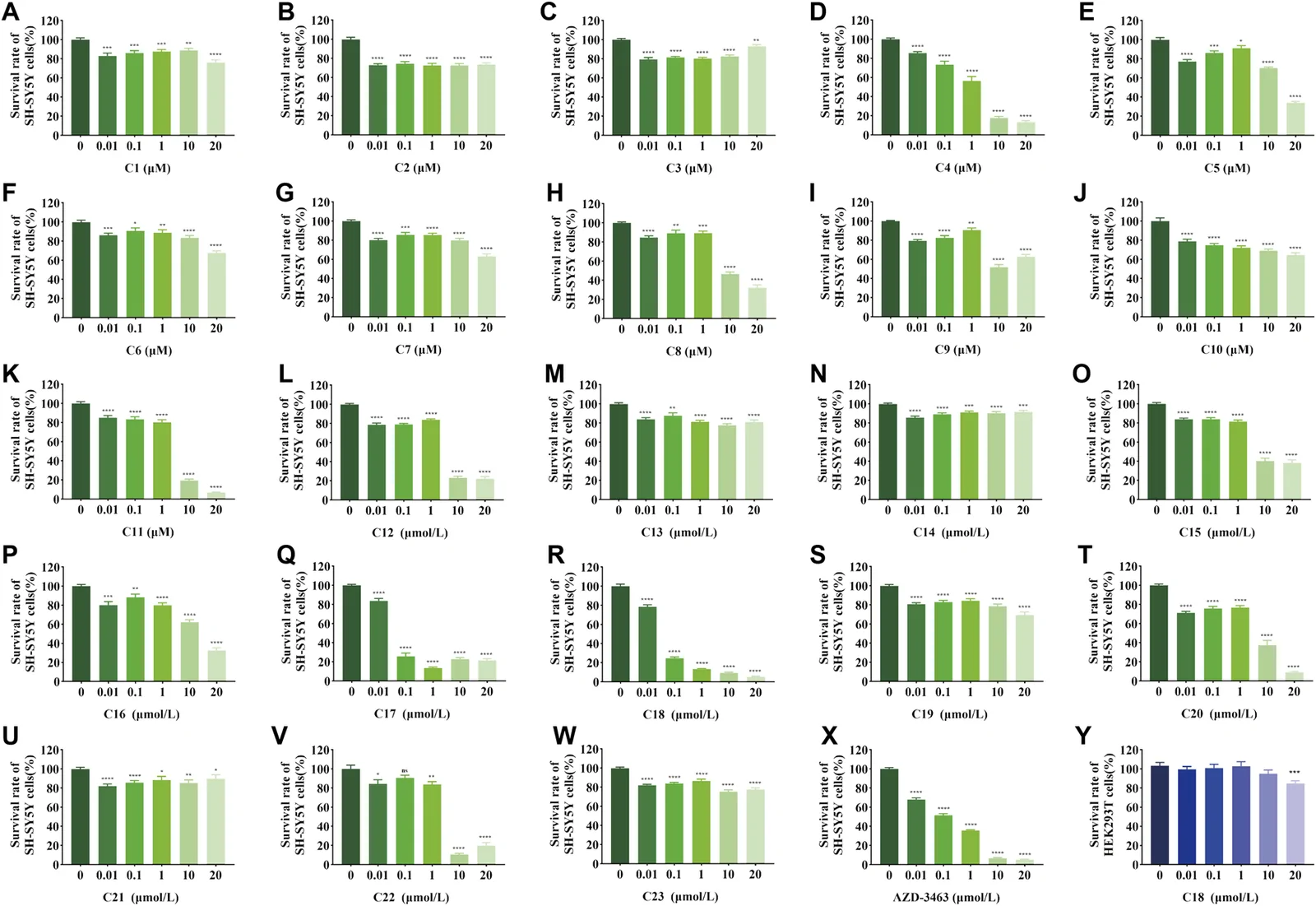
Effects of hit compounds on SH-SY5Y cell viability and selective toxicity of C18 in HEK293 T cells. **(A–X)** Survival rates of SH-SY5Y cells after 24 h treatment with gradient concentrations of the indicated hit compounds; **(Y)** Survival rates of HEK293T cells after 24 h treatment with gradient concentrations of C18. All statistical analyses were performed using the t-test, and data are presented as the mean ± SEM; *P < 0.05, **P < 0.01, ***P < 0.001, ****P < 0.0001 compared with the 0 group (*n* = 3).

To evaluate the anti-NB activity of the hit compounds, SH-SY5Y cells (ALK F1174L mutation) were first screened by CCK-8 assay (Figure 4). Eight compounds exhibited IC_50_ < 5 μM (Supplementary Table S6), with C18 showing the lowest IC_50_ of 0.03666 μM. To rule out non-specific cytotoxicity, C18 was tested on ALK-negative HEK293T cells (Figure 4Y): even at 20 μM, cell viability remained >80%, and an IC_50_ could not be fitted, confirming a wide *in vitro* safety window and ALK-dependent selectivity.

**FIGURE 5:**
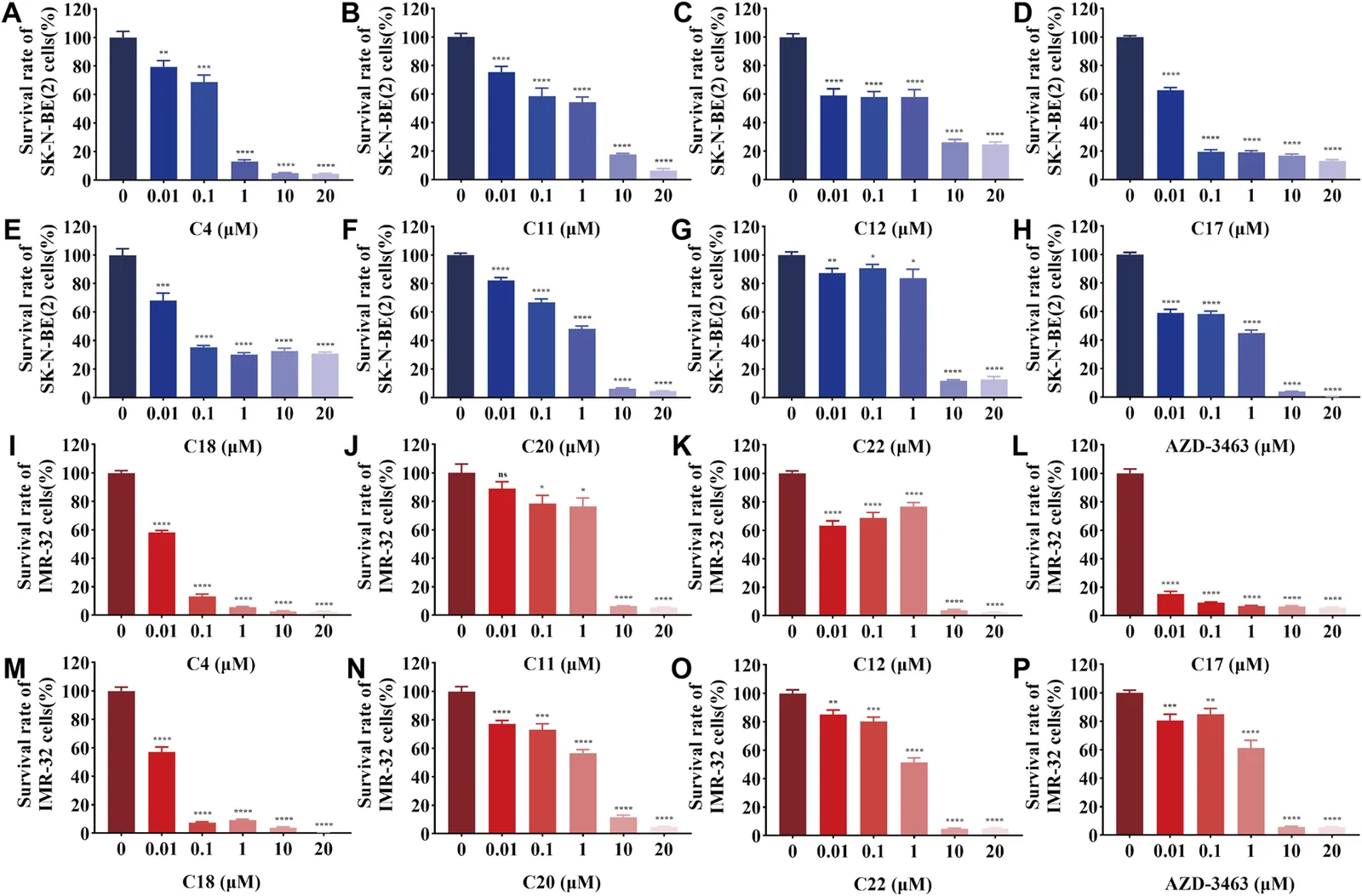
Effects of Preferred Compounds on Cell Viability of Different NB Cell Lines. **(A–H)** Survival rates of SK-N-BE (2) cells after 24 h treatment with gradient concentrations of C4 **(A)**, C11 **(B)**, C12 **(C)**, C17 **(D)**, C18 **(E)**, C20 **(F)**, C22 **(G)**, and the positive control AZD-3463 **(H)**. **(I–P)** Survival rates of IMR-32 cells after 24 h treatment with gradient concentrations of C4 **(I)**, C11 **(J)**, C12 **(K)**, C17 **(L)**, C18 **(M)**, C20 **(N)**, C22 **(O)**, and the positive control AZD-3463 **(P)**. All statistical analyses were performed using the t-test, and data are presented as the mean ± SEM; *P < 0.05, **P < 0.01, ***P < 0.001, ****P < 0.0001 compared with the 0 group (*n* = 3).

To further validate the active compounds beyond SH-SY5Y cells, two additional NB cell lines with distinct ALK activation mechanisms were selected: SK-N-BE (2) cells (MYCN amplification-driven ALK activation) and IMR-32 cells (ALK high expression). The results, shown in Figure 5 and Supplementary Table S6, demonstrated that C18 exhibited nanomolar IC_50_ values across both cell lines (0.04655 μM in SK-N-BE (2) and 0.01275 μM in IMR-32), confirming its broad anti-NB activity. Based on these findings, C18 was selected as the lead compound for subsequent mechanistic and *in vivo* studies.

### 3.5 Inhibitory effect of CDK12-IN-3 (C18) on proliferation, clonogenicity, and migration of NB cells

**FIGURE 6:**
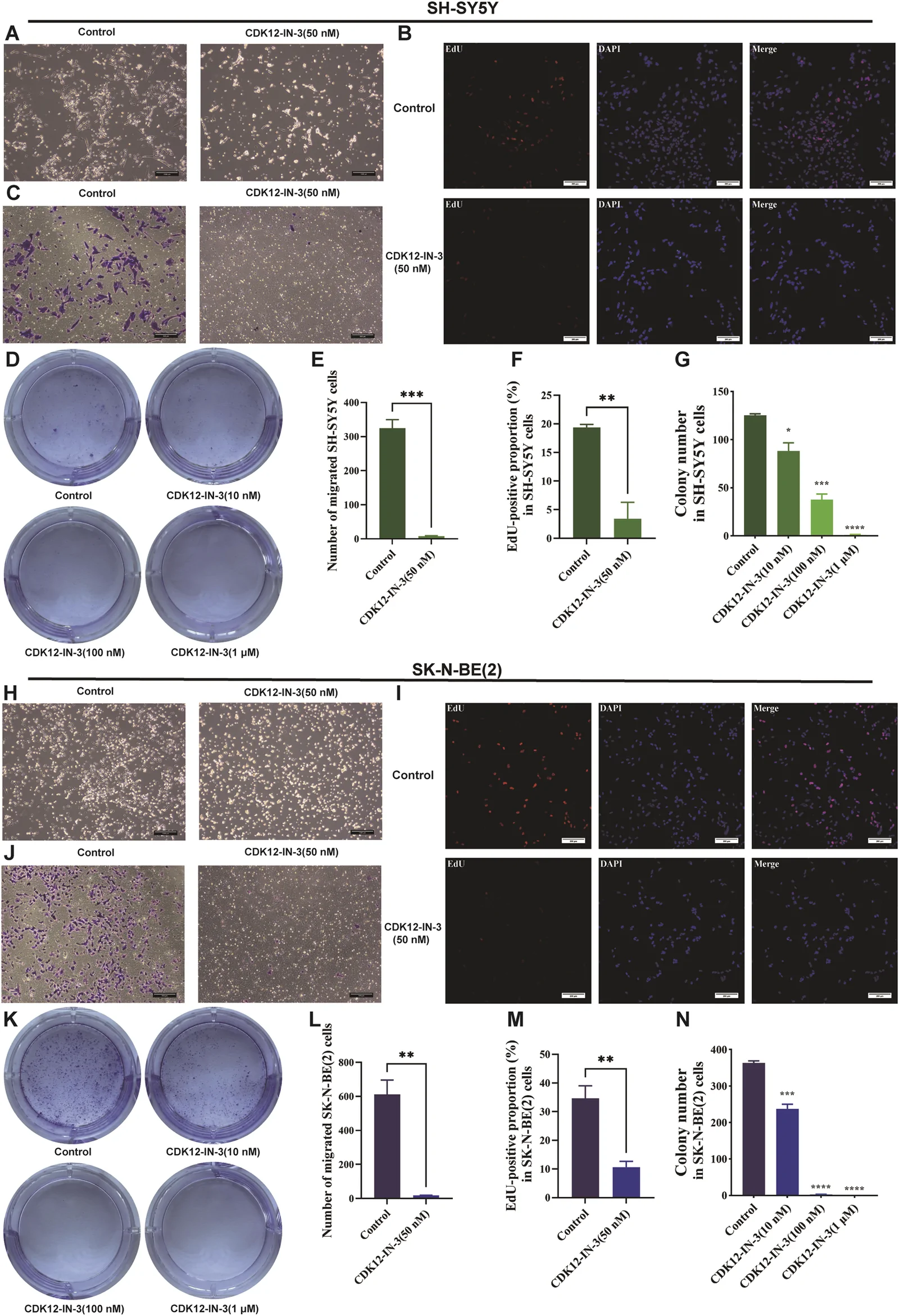
Inhibitory effects of CDK12-IN-3 (C18) on proliferation, colony formation and migration of neuroblastoma cells. **(A,H)** Morphological characteristics of SH-SY5Y **(A)** and SK-N-BE (2) **(H)** cells in the control group and those treated with 50 nM CDK12-IN-3 for 24 h were observed under a light microscope; **(B,I)** Fluorescence images of EdU proliferation assay (red: EdU-positive proliferating cells; blue: DAPI-stained nuclei), showing the differences in proliferation of SH-SY5Y **(B)** and SK-N-BE (2) **(I)** cells; **(C,J)** Crystal violet staining images of Transwell migration assay, showing the migration capacity of SH-SY5Y **(C)** and SK-N-BE (2) **(J)** cells; **(D,K)** Crystal violet staining results of colony formation assay, showing the colony formation ability of SH-SY5Y **(D)** and SK-N-BE (2) **(K)** cells (control group and treatment with 10 nM, 100 nM, 1 μM CDK12-IN-3); **(E,L)** Quantitative analysis of migration capacity of SH-SY5Y **(E)** and SK-N-BE (2) **(L)** cells; **(F,M)** Quantitative analysis of the proportion of EdU-positive cells in SH-SY5Y **(F)** and SK-N-BE (2) **(M)** cells; **(G,N)** Quantitative analysis of the number of formed colonies in SH-SY5Y **(G)** and SK-N-BE (2) **(N)** cells. All statistical analyses were performed using the t-test, and data are presented as the mean ± SEM; *P < 0.05, **P < 0.01, ***P < 0.001, ****P < 0.0001 compared with the control group (*n* = 3).

To further clarify the effects of CDK12-IN-3 (C18) on NB cells, the present study used SH-SY5Y and SK-N-BE (2) cells as research models to investigate its impacts on cell proliferation, colony formation, and migration capacities, with the results presented in Figure 6. Treatment with 50 nM CDK12-IN-3 for 24 h reduced cell density and altered morphology in both SH-SY5Y (Figure 6A) and SK-N-BE (2) cells (Figure 6H). EdU assay showed that 50 nM CDK12-IN-3 significantly reduced the proportion of EdU-positive cells in both SH-SY5Y (Figures 6B,F) and SK-N-BE (2) cells (Figures 6I,M). In the Transwell migration assay, crystal violet staining results showed that the number of migrated SH-SY5Y cells that traversed the chamber membrane was significantly fewer in the 50 nM CDK12-IN-3 (C18) treatment group than in the control group after 48 h (Figure 6C); quantitative analysis verified a significant inhibition of migration capacity (Figure 6E). A consistent tendency was observed in SK-N-BE (2) cells: the number of migrated cells in the CDK12-IN-3 (C18) treatment group decreased significantly (Figure 6J), and statistical results confirmed a notable suppression of migration ability (Figure 6L), demonstrating that this drug can attenuate the invasive and metastatic potential of NB cells. In the colony formation assay, crystal violet staining revealed that the number of colonies formed by SH-SY5Y cells decreased significantly with increasing concentrations of CDK12-IN-3 (C18) (10 nM, 100 nM, 1 μM) (Figure 6D); quantitative analysis confirmed a significant inhibition of colony formation ability (Figure 6G, showing a concentration-dependent inhibitory trend). SK-N-BE (2) cells presented a consistent pattern: the number of colonies in each CDK12-IN-3 (C18) treatment group was significantly lower than that in the control group, and the inhibitory effect was enhanced as the concentration increased (Figure 6K); statistical results indicated a marked reduction in colony formation capacity (Figure 6N), reflecting that this drug can concentration-dependently inhibit the *in vitro* proliferative potential of NB cells. In conclusion, 50 nM CDK12-IN-3 (C18) effectively inhibits the proliferation, migration, and colony formation capacities of both SH-SY5Y and SK-N-BE (2) NB cell lines, further validating its anti-NB activity.

### 3.6 Regulatory effects of CDK12-IN-3(C18) on ALK expression and apoptosis in NB cells

**FIGURE 7:**
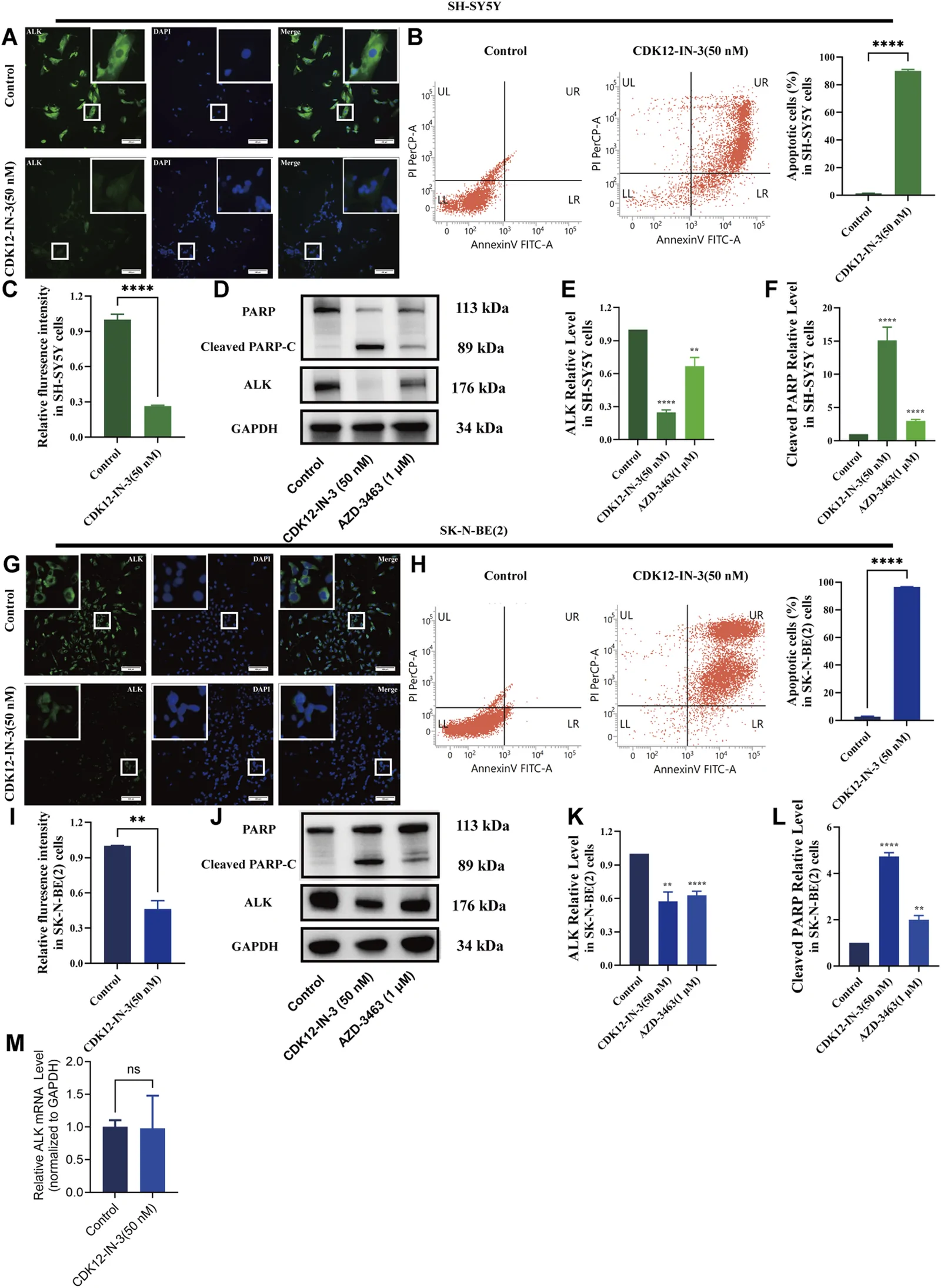
Effects of CDK12-IN-3 on ALK expression and apoptosis in NB SH-SY5Y and SK-N-BE (2) cells. SH-SY5Y cells: **(A)** IF staining (ALK: green; DAPI: nuclear staining, blue) showing the subcellular localization of ALK in the control group and 50 nM CDK12-IN-3 treatment group (including local magnified images); **(B)** Apoptosis detection by Annexin V/PI flow cytometry (left: scatter plots; right: statistical analysis of apoptotic rate, UR + LR representing apoptotic cells); **(C)** Quantitative analysis of ALK IF intensity; **(D)** Western blot analysis of PARP, Cleaved PARP, ALK and internal reference GAPDH protein expression; **(E,F)** Quantitative statistics of relative levels of ALK **(E)** and Cleaved PARP/PARP **(F)** (normalized to GAPDH). SK-N-BE (2) cells: **(G)** IF staining (same as **A**) showing the subcellular localization of ALK (including local magnified images); **(H)** Apoptosis detection by Annexin V/PI flow cytometry (same as **B**); **(I)** Quantitative analysis of ALK IF intensity; **(J)** Western blot analysis of protein expression (same as **D**); **(K,L)** Quantitative statistics of relative levels of ALK **(K)** and Cleaved PARP/PARP **(L)** (normalized to GAPDH). **(M)** qRT-PCR detection of relative ALK mRNA level normalized to GAPDH in SK-N-BE (2) cells. All statistical analyses were performed using the t-test, and data are presented as mean ± SEM (*n* = 3). **P < 0.01, ****P < 0.0001 vs. the control group.

To clarify the regulatory effects of CDK12-IN-3 on ALK expression and apoptosis in NB cells, experiments were performed using SH-SY5Y and SK-N-BE (2) cells as cellular models. In both SH-SY5Y and SK-N-BE (2) cells, 50 nM CDK12-IN-3 significantly reduced ALK fluorescence intensity (Figures 7A,C,G,I), increased the apoptotic rate (Figures 7B,H), downregulated ALK protein levels (Figures 7E,K), and elevated the Cleaved PARP/PARP ratio (Figures 7F,L). qRT-PCR analysis was further performed to detect the transcriptional level of ALK in SK-N-BE (2) cells. No statistically significant alteration of ALK mRNA abundance was observed following 50 nM CDK12-IN-3 treatment (Figure 7M), indicating that CDK12-IN-3-mediated ALK protein downregulation occurs at the post-transcriptional rather than transcriptional level. In summary, CDK12-IN-3 exerts anti-tumor effects in SH-SY5Y and SK-N-BE (2) NB cells by downregulating ALK expression and inducing cell apoptosis.

### 3.7 Validation of CDK12-IN-3 - ALK targeted binding: downstream pathway and direct binding

**FIGURE 8:**
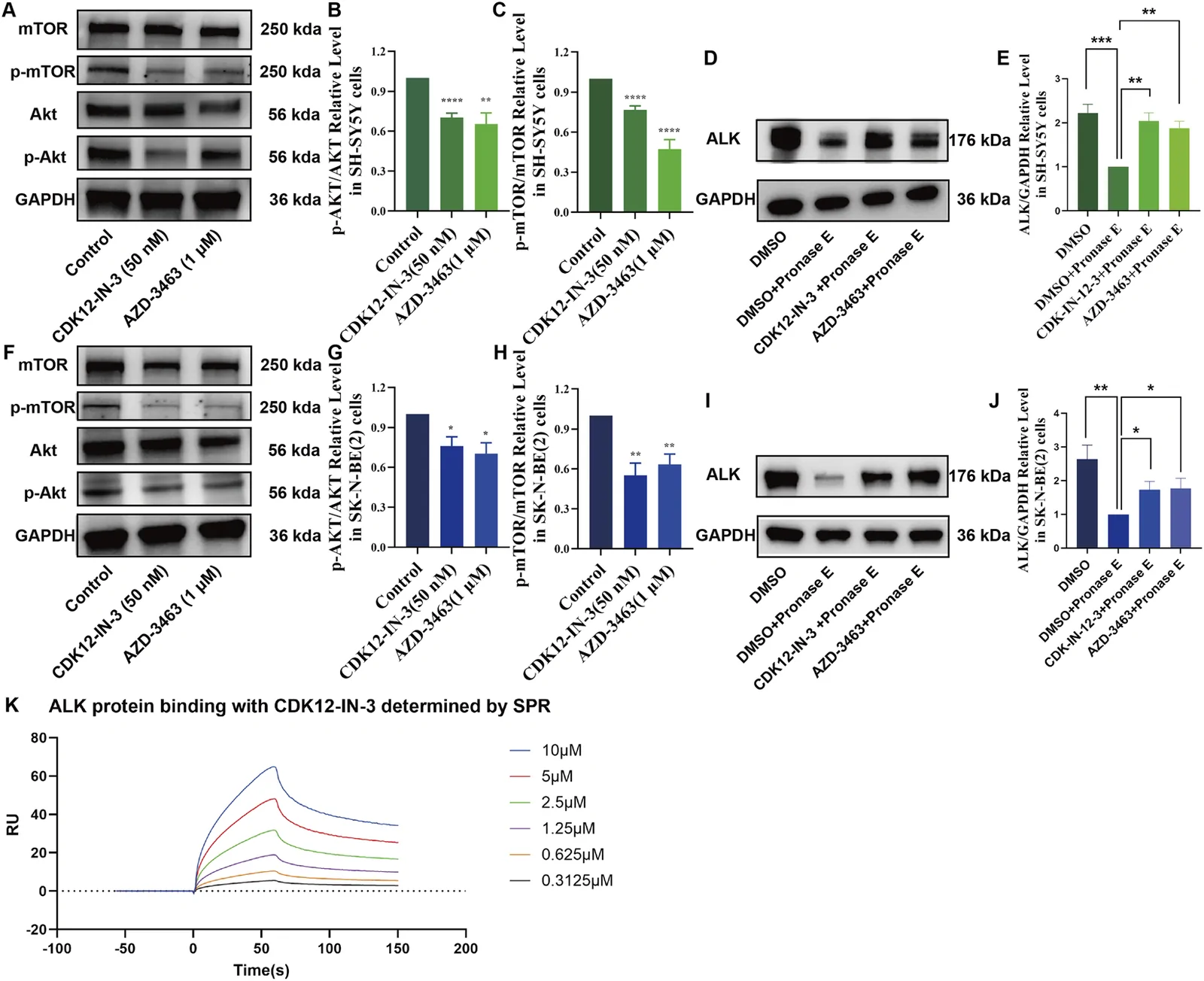
Validation of targeted binding between CDK12-IN-3 and ALK. **(A)** Western blot was performed to detect the protein expressions of mTOR, p-mTOR, Akt, p-Akt and the internal reference GAPDH in SH-SY5Y cells. The treatment groups were CDK12-IN-3 (50 nM) and AZD-3463 (1 μM); **(B,C)** Quantitative analysis of the relative levels of p-Akt/Akt **(B)** and p-mTOR/mTOR **(C)** in SH-SY5Y cells. Compared with the control group, **P < 0.01, ****P < 0.0001, (*n* = 3). **(D,E)** DARTS assay: Western blot **(D)** and quantitative analysis **(E)** were used to detect the protein stability of ALK following treatments with DMSO + Pronase E, CDK12-IN-3+Pronase E, or AZD-3463+Pronase E. Compared with the DMSO + Pronase E group, **P < 0.01, ***P < 0.001 (*n* = 3). **(F)** Western blot was conducted to detect the protein expressions of mTOR, p-mTOR, Akt, p-Akt and the internal reference GAPDH in SK-N-BE (2) cells, with the same treatment groups as in **A**; **(G,H)** Quantitative analysis of the relative levels of p-Akt/Akt **(G)** and p-mTOR/mTOR **(H)** in SK-N-BE (2) cells. Compared with the control group, *P < 0.05, **P < 0.01, (*n* = 3). **(I,J)** DARTS assay: Western blot **(I)** and quantitative analysis **(J)** were used to detect the protein stability of ALK under the three aforementioned treatments. Compared with the DMSO + Pronase E group, *P < 0.05, **P < 0.01 (*n* = 3). **(K)** SPR sensorgrams showing dose-dependent binding of CDK12-IN-3 to immobilized recombinant ALK protein.

To verify the binding between CDK12-IN-3 and ALK, AZD-3463 was used as a positive control. In SH-SY5Y cells, Western blot analysis revealed that treatment with CDK12-IN-3 (50 nM) or AZD-3463 (1 μM) significantly downregulated the relative levels of p-Akt/Akt and p-mTOR/mTOR compared with the control group, with identical trends of action between the two agents (Figures 8A–C). These findings were recapitulated in SK-N-BE (2) cells: treatment with CDK12-IN-3 (50 nM) led to a significant reduction in the relative levels of p-Akt/Akt and p-mTOR/mTOR, which was highly consistent with the activity profile of the positive control AZD-3463 (Figures 8F–H). As a canonical downstream pathway of ALK, the inhibitory phenotype of Akt/mTOR directly indicated the regulatory effect of CDK12-IN-3 on ALK activity.

To directly confirm the interaction between CDK12-IN-3 and ALK, the DARTS assay was employed. In SH-SY5Y cells, compared with the DMSO + Pronase E control group, the CDK12-IN-3+Pronase E treatment group exhibited significantly enhanced ALK protein degradation resistance and elevated relative levels of ALK/GAPDH, with a phenotype identical to that of the AZD-3463 treatment group, directly confirming the binding of CDK12-IN-3 to ALK (Figures 8D,E). DARTS assays in SK-N-BE (2) cells further consolidated this conclusion: the ALK degradation-resistant phenotype and ALK/GAPDH quantitative results in the CDK12-IN-3 treatment group were highly consistent with those in the AZD-3463 group (Figures 8I,J). To further quantitatively validate the direct physical interaction between CDK12-IN-3 and ALK at the biophysical level, SPR assay was performed using purified recombinant ALK protein. As shown in Figure 8K, CDK12-IN-3 exhibited dose-dependent binding to immobilized ALK protein, with sensorgrams showing typical association and dissociation curves. Global fitting of the kinetic data to a 1:1 Langmuir binding model yielded an equilibrium dissociation constant (*K*_D_) of 5.33 μM, providing direct quantitative evidence for the direct binding between CDK12-IN-3 and ALK.

### 3.8 Therapeutic efficacy of CDK12-IN-3 in a nude mouse xenograft model of NB cells

**FIGURE 9:**
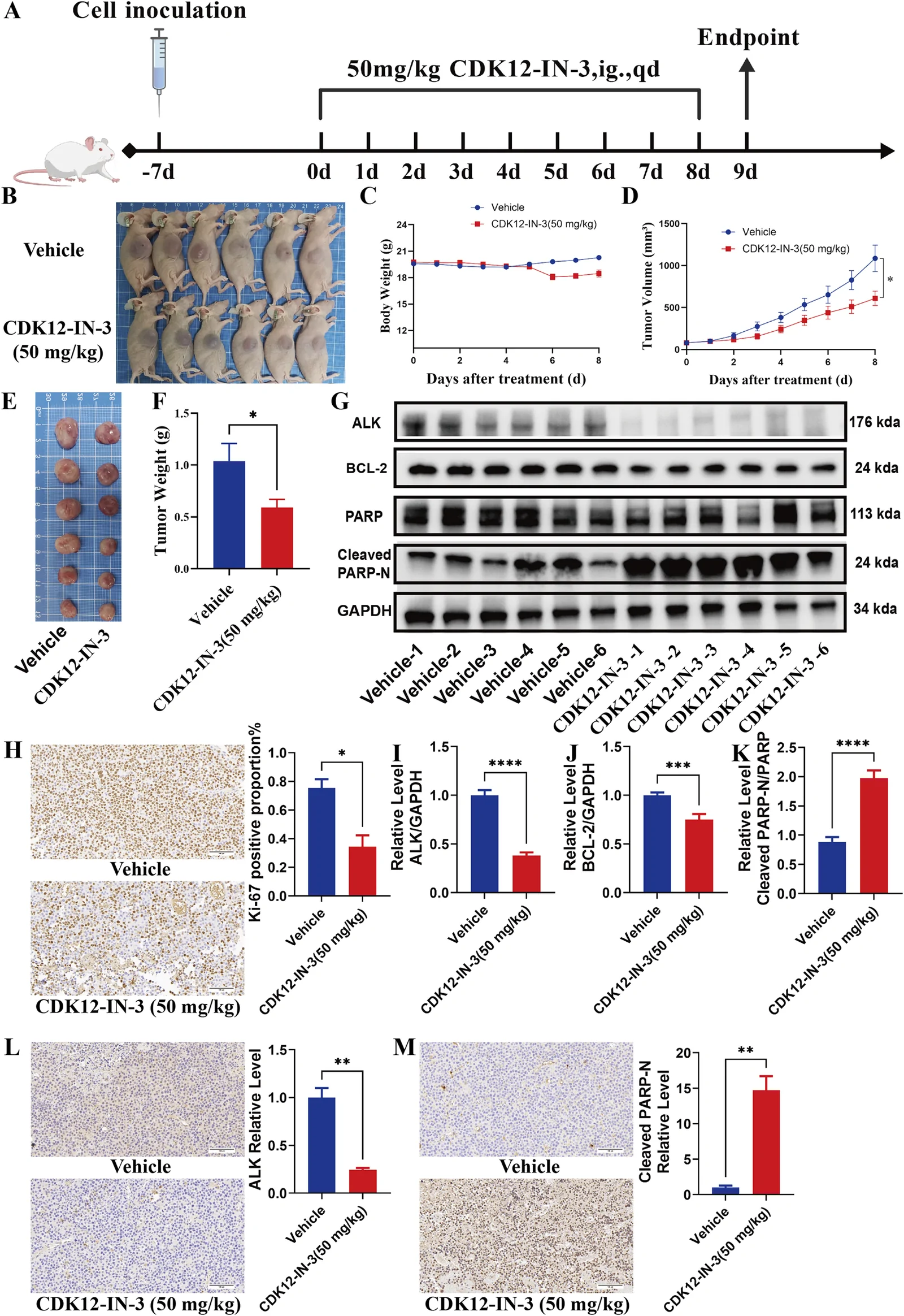
Therapeutic efficacy of CDK12-IN-3 in a nude mouse xenograft model of NB cells. **(A)** Schematic diagram of the *in vivo* experimental procedure; **(B)** Comparison of the appearance of mice between the Vehicle group and CDK12-IN-3 (50 mg/kg) group at the experimental endpoint (Day 9); **(C)** Dynamic curve of mouse body weight over the treatment days; **(D)** Dynamic curve of tumor volume over the treatment days; **(E)** Representative images of tumors from the Vehicle group and CDK12-IN-3 group at the experimental endpoint (Day 9); **(F)** Quantitative statistics of tumor weight at the experimental endpoint (Day 9), (*n* = 6); **(G)** Western blot analysis of the protein expression of ALK, BCL-2, PARP, Cleaved PARP-N, and the internal reference GAPDH in tumor tissues, with quantitative statistics of the relative protein levels of ALK **(I)**, BCL-2 **(J)**, and Cleaved PARP-N/PARP **(K)** (*n* = 6); **(H)** Ki-67 IHC staining (left) and quantitative statistics of the positive proportion (right) (*n* = 3); **(L)** ALK IHC staining (left) and quantitative statistics of the relative level (right) (*n* = 3); **(M)** Cleaved PARP-N IHC staining (left) and quantitative statistics of the relative level (right) (*n* = 3). All statistical analyses were performed using the t-test, and data are presented as the mean ± SEM; *P < 0.05, **P < 0.01, ***P < 0.001, ****P < 0.0001 compared with the Vehicle group.

To evaluate the *in vivo* therapeutic efficacy of CDK12-IN-3 against NB, a nude mouse xenograft model of NB cells was established in this study. The detailed experimental procedure was as follows: nude mice were subcutaneously implanted with the murine NB cell line Neuro-2a on Day −7. On Day 0, after measuring tumor volumes, mice were randomly divided into the vehicle control group (Vehicle group) and CDK12-IN-3 treatment group (50 mg/kg) using a normal distribution equidistant grouping method, with six mice per group, to ensure consistent baseline tumor volumes across groups. Starting from Day 0, mice in the treatment group were administered 50 mg/kg CDK12-IN-3 via daily oral gavage, while those in the Vehicle group received an equal volume of vehicle. During the treatment period, the body weight and tumor volume of mice in both groups were monitored and recorded daily, and oral gavage administration was continued until the experimental endpoint on Day 9 (Figure 9A). At the endpoint, tumor-bearing mice were anesthetized with isoflurane, and their general appearance was photographed to document their physical status. Subsequently, tumor tissues were excised and photographed for recording. Representative appearances of mice in the Vehicle group and CDK12-IN-3 (50 mg/kg) group at the experimental endpoint are shown in Figure 9B.

During the experiment, continuous monitoring of mouse body weight revealed that the body weight change trends of the CDK12-IN-3 treatment group were basically consistent with those of the Vehicle control group, without significant decrease or abnormal fluctuation (Figure 9C). This indicates no significant systemic toxicity at the tested dose. Tumor growth differed significantly between groups: the tumor volume growth rate in the CDK12-IN-3 group was markedly slower than in the control group, and this difference widened over time (Figure 9D). At the experimental endpoint, the gross tumor size of the treatment group was significantly smaller than that of the control group (Figure 9E), and tumor weight measurements also showed that the tumor weight was significantly reduced compared with the control group (Figure 9F). These results demonstrate that CDK12-IN-3 can effectively inhibit the growth of NB xenografts *in vivo*.

To further explore the underlying mechanism, molecular-level detections were performed: in terms of proliferation inhibition, Ki-67 immunohistochemical (IHC) staining results showed that the proportion of Ki-67-positive cells in tumor tissues of the CDK12-IN-3 treatment group was significantly lower than that of the control group (Figure 9H), suggesting that the proliferative activity of tumor cells was significantly inhibited. Meanwhile, Western blot analysis revealed that the expression level of ALK protein in tumor tissues of the treatment group was significantly downregulated (Figures 9G,I), and IHC staining also showed a significant reduction in the positive signal intensity of ALK (Figure 9L), indicating that CDK12-IN-3 may inhibit the proliferation of NB cells by downregulating the ALK signaling pathway. In terms of apoptosis induction, Western blot results showed that the expression level of the anti-apoptotic protein BCL-2 in the treatment group was significantly decreased (Figures 9G,J), while the expression level of the apoptosis-related marker Cleaved PARP-N was significantly increased (Figures 9G,K). IHC staining further confirmed that the positive signal of Cleaved PARP-N in tumor tissues of the treatment group was significantly enhanced (Figure 9M), suggesting that CDK12-IN-3 can induce apoptosis of NB cells *in vivo*. In summary, CDK12-IN-3 exhibits significant antitumor efficacy in the NB xenograft model, likely through inhibiting ALK-mediated proliferation and inducing apoptosis. Moreover, it shows good *in vivo* safety at the experimental dose, providing important *in vivo* experimental evidence for its subsequent clinical translation research.

## 4 Discussion

This study integrated virtual screening with multi-dimensional MD simulations and drug repurposing to identify CDK12-IN-3 as a potential ALK inhibitor for NB, with *in vitro* and *in vivo* validation of its pharmacodynamic effects. Drug repurposing is an efficient strategy for anti-tumor drug development, particularly for rare diseases such as NB (El Kababri et al., 2020; Liu et al., 2016), as it can alleviate the long development cycles, high costs, and high failure rates of traditional drug discovery. Instead of adopting *de novo* drug design, this study screened 7,322 compounds from four functionally complementary compound libraries; among these efforts, MD simulations played a role in improving screening accuracy — initially expanding the screening dimension from “static structural matching” to “dynamic binding stability assessment”. Traditional virtual screening methods (e.g., HTVS, SP/XP) mostly rely on the static structural complementarity between ligands and the ALK binding pocket. In contrast, this study performed 100 ns dynamic behavior simulations of ALK (wild-type/mutant)-ligand complexes under simulated physiological conditions via MD simulations, quantitatively analyzing indicators such as ΔG_bind_, ALK backbone RMSD, and ligand RMSD. This approach eliminated some false-positive compounds that exhibited excellent static docking scores but unstable dynamic binding to a certain extent. For NB, which has high genetic heterogeneity and relatively limited therapeutic options, this MD simulation-assisted drug repurposing method may provide a feasible exploratory pathway for the development of targeted therapeutic drugs for high-risk/relapsed NB.

The identification of CDK12-IN-3 as an ALK inhibitor through drug repurposing offers several distinct therapeutic advantages over existing clinical ALK inhibitors. First, CDK12-IN-3 possesses a structurally distinct scaffold unrelated to the crizotinib–ceritinib–alectinib–lorlatinib chemical lineage. In the lorlatinib clinical trial, compound mutations such as F1174L/G1202R and F1174L/L1196M conferred 10- to 50-fold resistance to lorlatinib (Berko et al., 2023); a structurally unrelated compound like CDK12-IN-3 may retain activity against these resistant variants, although this requires experimental validation. Second, unlike all currently approved ALK inhibitors that function exclusively as ATP-competitive kinase inhibitors, CDK12-IN-3 exhibits a dual mechanism of action—direct kinase binding (KD = 5.33 μM by SPR) and concurrent downregulation of ALK protein expression. This dual action may reduce the likelihood of resistance emergence, as tumor cells would need to simultaneously bypass both kinase inhibition and protein degradation pathways. Third, CDK12-IN-3 demonstrated nanomolar-level cytotoxicity (IC_50_ = 12.75–46.55 nM) across three genetically distinct NB cell lines, significantly outperforming the positive control AZD-3463 (IC_50_ = 100–1,282 nM), while showing no significant toxicity to ALK-negative HEK293T cells even at 20 μM. This wide *in vitro* safety window is particularly noteworthy given that lorlatinib treatment is associated with a 90% incidence of hypertriglyceridemia and substantial CNS toxicity in adult patients (Goldsmith et al., 2023). Fourth, computational selectivity analysis across 10 homologous tyrosine kinases revealed a ΔΔGbind ≥1.76 kcal/mol in favor of ALK, suggesting that CDK12-IN-3 may offer improved target selectivity compared to multi-kinase inhibitors such as entrectinib, which concurrently targets ALK, TRKA/B/C, and ROS1 (Pacenta and Macy, 2018). Finally, the *in vivo* efficacy of CDK12-IN-3 (50 mg/kg, oral administration) in suppressing NB xenograft growth without observable systemic toxicity provides a strong rationale for further preclinical development. Collectively, these findings position CDK12-IN-3 as a structurally and mechanistically novel ALK inhibitor candidate that addresses several unmet needs associated with current clinical ALK inhibitors in NB.

This study conducted five independent MD simulations with random initial velocities for each ALK-ligand complex, which was one of the fundamental attempts to ensure the scientific validity and reliability of the results (D et al., 2019; Deng et al., 2013). However, five replicate simulations indicated that the binding stability of CDK12-IN-3 to ALK (e.g., convergent RMSD and consistent ΔG_bind_) may be a universal characteristic rather than a random result.

The SMD and US simulations in this study quantified the dissociation free energy (ΔG) of each ligand from the ALK binding pocket. The ΔG value of the ALK-CDK12-IN-3 complex was close to that of the positive control AZD-3463, suggesting high binding stability; in contrast, the ΔG of ALK (wild-type)-C20 was only 11.19 kJ/mol, significantly lower than the positive control, and was therefore excluded from subsequent validation.

To comprehensively validate the specific binding between CDK12-IN-3 and ALK, this study employed a multi-dimensional experimental approach integrating Western blot analysis of downstream signaling, DARTS assay, and SPR-based direct binding measurement. The results demonstrated that CDK12-IN-3 significantly downregulated ALK protein expression, suppressed the phosphorylation of its downstream PI3K/AKT/mTOR pathway, and enhanced ALK protein stability against proteolytic degradation, collectively confirming the specific engagement of CDK12-IN-3 with ALK in the cellular context. Furthermore, SPR analysis using a Biacore system with immobilized recombinant ALK kinase domain protein yielded a KD value of 5.33 μM for the CDK12-IN-3-ALK interaction, with well-fitted 1:1 Langmuir binding kinetics. Although this KD value is higher than those of typical clinical ALK inhibitors (low nanomolar range), low micromolar affinities determined by SPR are widely accepted as evidence of specific binding in early-stage drug discovery (Patching, 2014; Shepherd et al., 2022). It is noteworthy that the KD value measured by SPR (5.33 μM) appears discrepant from the cellular effective concentration (∼50 nM) at which CDK12-IN-3 exerts anti-NB activity. This discrepancy may be attributed to several factors. First, the SPR assay utilized a recombinant ALK kinase domain (32 kDa) rather than full-length ALK protein; the absence of juxtamembrane, transmembrane, and extracellular regulatory domains may affect the overall conformational integrity and binding pocket accessibility of ALK, potentially leading to reduced measured affinity (Roskoski, 2024). Second, the recombinant ALK protein used for SPR was likely in an inactive or basal conformation, whereas in the cellular environment, ALK exists in a dynamic equilibrium between active and inactive states, and the active conformation may exhibit higher affinity for ATP-competitive inhibitors (Arter et al., 2022). Third, the intracellular milieu contains co-factors, scaffolding proteins, and post-translational modifications that may stabilize the drug-target interaction, resulting in enhanced apparent potency at the cellular level. Collectively, the multi-layered experimental evidence supports that CDK12-IN-3 specifically binds to ALK, and the moderate SPR KD value does not contradict its potent cellular activity but rather reflects inherent differences between the cell-free biochemical assay and the more complex cellular environment.

Several limitations related to the evaluation of target selectivity in this study are objectively elaborated as follows. First, no *in vitro* kinase profiling assays were performed in this work. Instead, MM-PBSA binding free energy calculations based on multiple independent molecular dynamics simulations were adopted to evaluate the binding preference of C18 toward ALK over ten homologous tyrosine kinases. Although computational simulation approaches cannot fully replace experimental kinase selectivity screening, the MM-PBSA method has been widely recognized as an effective tool for assessing ligand binding propensity in early-stage drug discovery (Genheden and Ryde, 2015). The ΔΔGbind between wild-type ALK and TRKB, the off-target kinase with the closest binding affinity, was 1.76 kcal/mol. Calculated via the fundamental thermodynamic formula ΔΔG = −RT ln (KD_1_/KD_2_), this energy gap indicates that the compound binds ALK approximately 17-fold tighter than TRKB. For most of the remaining tested kinases, the energy differences ranged from 4 to 6 kcal/mol, corresponding to binding selectivity of roughly 700 to 20,000-fold. These semi-quantitative computational results verify the preferential binding of this compound to ALK. Second, previous literature has reported that CDK12-IN-3 (Compound 7) acts as a selective CDK12 inhibitor, with an enzymatic IC_50_ of 491 nM against the CDK12/cyclin K complex (Johannes et al., 2018). Nevertheless, multiple lines of evidence demonstrate that the anti-neuroblastoma activity observed at the low nanomolar concentrations used in this study is not primarily driven by CDK12 inhibition. In CDK12-dependent cellular models such as OV90 ovarian cancer cells, a concentration of approximately 100 nM is required to markedly suppress CDK12 downstream signaling (RNA polymerase II Ser2 phosphorylation) and block cell proliferation (Johannes et al., 2018). In contrast, CDK12-IN-3 exerted potent cytotoxicity against neuroblastoma cells, with IC_50_ values of 36.66 nM for SH-SY5Y cells and merely 12.75 nM for IMR-32 cells, which are only 1/3 to 1/8 of the dosage required to elicit CDK12-related cellular phenotypes. A concentration of 50 nM was utilized for mechanistic assays including EdU proliferation tests, Transwell migration assays and Western blot analysis. This dosage is below the cellular threshold for effective CDK12 inhibition and is insufficient to induce robust CDK12 binding and suppression. Consistent with this observation, the compound exhibited no obvious cytotoxicity against ALK-negative HEK293T cells across the same concentration gradients, revealing that its anti-proliferative effect relies on ALK expression rather than non-specific broad cytotoxicity or CDK12 inhibition. Furthermore, CDK12-IN-3 persistently downregulated ALK protein levels and repressed the ALK downstream PI3K/AKT/mTOR signaling cascade. DARTS target capture experiments further provided direct evidence for the specific physical interaction between CDK12-IN-3 and ALK protein. Collectively, these mutually corroborating findings confirm that the anti-neuroblastoma effect of CDK12-IN-3 at low nanomolar concentrations is predominantly mediated by ALK inhibition. Even so, systematic *in vitro* kinase profiling assays are still required to fully characterize the target specificity of this compound, and such experiments will be implemented in our future research.

This study has several limitations: ① Model limitations: only 4 NB cell lines were used at the cellular level, while NB has high genetic heterogeneity; the pharmacodynamic effects of CDK12-IN-3 in more diverse cell lines need further verification. At the animal level, the nude mouse subcutaneous xenograft model of Neuro-2a cells cannot simulate the tumor microenvironment of orthotopic NB tumors or bone marrow metastasis, which differs from clinical reality to a certain extent. ② Simulation-related limitations: 100 ns MD simulations can only evaluate short-term binding stability; longer time-scale simulations may reveal more subtle conformational changes in ALK. The MM/PBSA method for calculating ΔG_bind_ did not include the entropy term, which may introduce a certain bias to the assessment of binding free energy. In addition, the study only focused on two common ALK mutations (F1174L, R1275Q) in NB, and did not cover some clinically common drug-resistant mutations, so the anti-resistance potential of CDK12-IN-3 cannot be fully evaluated for the time being.

Based on the above limitations, future research can further explore the following directions: ① Optimize MD simulation strategies: attempt to extend the simulation time scale to the microsecond level and adopt enhanced sampling techniques to capture rare conformational changes of ALK; include the entropy term in MM/PBSA calculations to improve the accuracy of binding free energy assessment; expand the range of ALK mutations to screen compounds with broader-spectrum activity. ② Improve *in vivo*/*in vitro* models: construct NB orthotopic tumor models and metastasis models that are more clinically relevant; evaluate the pharmacodynamic effects of CDK12-IN-3 in NB cell lines with different genetic backgrounds to clarify its potential applicable population; explore combination regimens with other therapeutic approaches to enhance anti-tumor efficacy. ③ Promote the research framework: extend the technical framework of “virtual screening-MD/SMD/US - *in vivo*/*in vitro* validation” established in this study to the development of targeted drugs for other childhood malignant solid tumors, with the aim of improving the R&D efficiency of drugs for rare childhood tumors.

## 5 Conclusion

This study aimed to address the issues of drug resistance to ALK inhibitors and low efficiency of traditional new drug development in NB. By integrating virtual screening with multi-dimensional MD simulation techniques, potential ALK inhibitors were identified and validated through a drug repurposing strategy.

After multiple rounds of screening of 7,322 compounds and verification via MD/SMD/US simulations, CDK12-IN-3 was confirmed to have high binding affinity for wild-type ALK as well as F1174L and R1275Q mutant ALK. In three NB cell lines including SH-SY5Y and SK-N-BE (2), it significantly inhibited cell proliferation, migration, and colony formation, exerting its effects by downregulating ALK expression, suppressing the PI3K/AKT/mTOR pathway, and inducing apoptosis. Nude mouse xenograft models further verified its remarkable *in vivo* anti-tumor activity with no obvious systemic toxicity.

The “dynamic simulation-assisted drug repurposing” technical framework established in this study expands the screening dimension from static structural matching to dynamic binding stability assessment, providing an efficient paradigm for the development of targeted drugs for high-risk/relapsed NB and other rare pediatric tumors. Despite limitations in the diversity of cell models, simulation time scale, and coverage of ALK mutations, the preliminary validation of CDK12-IN-3 lays a foundation for subsequent structural optimization, mechanism exploration, and clinical translation. Meanwhile, the related technical system offers a reference for the research and development of precision therapies for rare tumors.

## Data Availability

The original contributions presented in the study are publicly available. This data can be found here: https://github.com/bookasiap/li-zhong/tree/main/ALK.
