# Supplementary material for: Virtual screening and MD simulation-guided discovery of CDK12-IN-3, a potent ALK inhibitor suppressing neuroblastoma growth in vitro and in vivo
Source: Front Chem. 2026 Aug 10;14:1870293. doi: 10.3389/fchem.2026.1870293 (PMC13530976; doi:10.3389/fchem.2026.1870293)
Supplement: Supplementary file 1 [file DataSheet1.docx]

**FIGURE S1. Backbone RMSD Profiles of ALK-Hit Compound Complexes and Positive Control Over 100 ns MD Simulation.**

**Figure S2. 3D FEL constructed from the 50-100 ns equilibrium trajectory of 100 ns MD simulations for ligand-ALK(Wild-Type) complexes. Each panel shows the 3D FEL surface graph and the two-dimensional ligand-protein interaction diagram of the representative binding conformation extracted from the lowest free-energy minimum. (A) C4-ALK(Wild-Type) complex; (B) C15-ALK(Wild-Type) complex; (C) C18-ALK(Wild-Type) complex; (D) C20-ALK(Wild-Type) complex; (E) C24-ALK(Wild-Type) complex.**

**Figure S3. 3D FEL constructed from the 50-100 ns equilibrium trajectory of 100 ns MD simulations for ligand-ALK(F1174L) complexes. Each panel shows the 3D FEL surface graph and the two-dimensional ligand-protein interaction diagram of the representative binding conformation extracted from the lowest free-energy minimum. (A) C4-ALK(F1174L) complex; (B) C15-ALK(F1174L) complex; (C) C18-ALK(F1174L) complex; (D) C20-ALK(F1174L) complex; (E) C24-ALK(F1174L) complex.**

**Figure S4. 3D FEL constructed from the 50-100 ns equilibrium trajectory of 100 ns MD simulations for ligand-ALK(R1275Q) complexes. Each panel shows the 3D FEL surface graph and the two-dimensional ligand-protein interaction diagram of the representative binding conformation extracted from the lowest free-energy minimum. (A) C4-ALK(R1275Q) complex; (B) C15-ALK(R1275Q) complex; (C) C18-ALK(R1275Q) complex; (D) C20-ALK(R1275Q) complex; (E) C24-ALK(R1275Q) complex.**

**Table S1. Catalog numbers and purities of hit compounds and positive control drugs**

| **Compound** | **Cat. No.** | **Purity** | **Compound** | **Cat. No.** | **Purity** |
| --- | --- | --- | --- | --- | --- |
| Emodin | HY-14393 | 99.25% | Onatasertib | HY-16956 | 96.20% |
| Chrysophanol | HY-13595 | 98.80% | CC-401 | HY-13022A | 98.60% |
| Physcion | HY-N0108 | 98.53% | [UNC2025](https://www.ncbi.nlm.nih.gov/pcsubstance/?term="UNC2025"[CompleteSynonym] AND 73425588[StandardizedCID]" \o "" \t "https://pubchem.ncbi.nlm.nih.gov/compound/_blank) | HY-12344 | 99.83% |
| Mitoxantrone | HY-13502 | 98.70% | KW-2449 | HY-10339 | 98.45% |
| (R)-Roscovitine | HY-30237 | 99.53% | Dinaciclib | HY-10492 | 99.64% |
| Fisetin | HY-N0182 | 98.03% | CDK12-IN-3 | HY-112261 | 99.86% |
| Morin | HY-N0621 | 97.02% | Voruciclib | HY-12422 | 99.77% |
| Cvt-313 | HY-15339 | 99.90% | LDC4297 | HY-12653 | 98.25% |
| APY29 | HY-17537 | 99.21% | LX7101 | HY-12659 | 99.54% |
| PHA-793887 | HY-11001 | 99.25% | Purvalanol A | HY-18299A | 98.30% |
| BS-181 hydrochloride | HY-13266A | 99.80% | Ca2+ channel agonist 1 | HY-41076 | 99.71% |
| AZ3146 | HY-14710 | 98.00% | AZD-3463 | [HY-15609](https://www.medchemexpress.cn/azd-3463.html) | 99.58% |

**Table S2. ADME Parameter Values of Hit Compounds and Positive Controls.**

| **S.no** | **Molecular weight** | **TPSA**  **(Å²)** | **H-bond donors** | **H-bond acceptors** | **Consensus Log P_o/w_** | **Log S (ESOL)** | **PAINS** |
| --- | --- | --- | --- | --- | --- | --- | --- |
| C1 | 270.24 | 94.83 | 3 | 5 | 1.88 | -3.67 | quinone_A |
| C2 | 254.24 | 74.6 | 2 | 4 | 2.42 | -4.11 | quinone_A |
| C3 | 284.26 | 83.83 | 2 | 5 | 2.23 | -3.87 | quinone_A |
| C4 | 444.48 | 163.18 | 8 | 8 | 0.1 | -2.71 | anthranil_one_A, quinone_A |
| C5 | 354.45 | 87.89 | 3 | 4 | 2.1 | -3.93 | None |
| C6 | 286.24 | 111.13 | 4 | 6 | 1.07 | -2.99 | catechol_A |
| C7 | 302.24 | 131.36 | 5 | 7 | 0.79 | -3.19 | None |
| C8 | 400.47 | 108.56 | 3 | 6 | 0.85 | -3.07 | None |
| C9 | 332.36 | 107.2 | 4 | 4 | 2.62 | -3.88 | None |
| C10 | 361.48 | 81.33 | 2 | 4 | 1.49 | -2.44 | None |
| C11 | 416.99 | 80.27 | 3 | 3 | 3.77 | -5.15 | None |
| C12 | 452.55 | 86.44 | 1 | 6 | 1.48 | -5.29 | None |
| C13 | 397.47 | 100.47 | 2 | 6 | 1.24 | -3.05 | None |
| C14 | 388.47 | 82.72 | 2 | 5 | 3.49 | -4.75 | None |
| C15 | 476.66 | 69.45 | 2 | 5 | 3.14 | -5.11 | None |
| C16 | 332.4 | 61.02 | 2 | 3 | 2.62 | -3.76 | None |
| C17 | 396.49 | 91.15 | 2 | 4 | 1.39 | -3.42 | None |
| C18 | 470.52 | 107.78 | 3 | 7 | 2.92 | -4.84 | None |
| C19 | 469.84 | 94.14 | 3 | 9 | 3.38 | -4.97 | None |
| C20 | 432.52 | 94.19 | 2 | 6 | 1.88 | -4.68 | None |
| C21 | 451.52 | 129.47 | 3 | 6 | 1.66 | -3.71 | None |
| C22 | 388.89 | 87.89 | 3 | 4 | 3.03 | -4.9 | None |
| C23 | 354.45 | 87.89 | 3 | 4 | 2.03 | -3.94 | None |
| C24 | 448.95 | 92.09 | 3 | 4 | 3.55 | -5.43 | None |

**Table S3. Predicted Toxicity Parameter Values of Hit Compounds and Positive Controls.**

| **S.no** | **SMILES** | **Hepatotoxicity** | **Mutagenicity** | **Oral toxicity** |
| --- | --- | --- | --- | --- |
| C1 | CC1=CC2=C(C(=C1)O)C(=O)C3=C(C2=O)C=C(C=C3O)O | Inactive(0.70) | Active(0.93) | Ⅴ(70.97%) |
| C2 | CC1=CC2=C(C(=C1)O)C(=O)C3=C(C2=O)C=CC=C3O | Inactive(0.71) | Active(0.99) | Ⅴ(70.97%) |
| C3 | CC1=CC2=C(C(=C1)O)C(=O)C3=C(C2=O)C=C(C=C3O)OC | Inactive(0.71) | Active(0.94) | Ⅴ(69.26%) |
| C4 | C1=CC(=C2C(=C1NCCNCCO)C(=O)C3=C(C=CC(=C3C2=O)O)O)NCCNCCO | Inactive(0.84) | Active(0.89) | Ⅳ(100%) |
| C5 | CC[C@H](CO)NC1=NC(=C2C(=N1)N(C=N2)C(C)C)NCC3=CC=CC=C3 | Inactive(0.76) | Inactive(0.51) | Ⅲ(68.07%) |
| C6 | C1=CC(=C(C=C1C2=C(C(=O)C3=C(O2)C=C(C=C3)O)O)O)O | Inactive(0.70) | Inactive(0.53) | Ⅲ(72.90%) |
| C7 | C1=CC(=C(C=C1O)O)C2=C(C(=O)C3=C(C=C(C=C3O2)O)O)O | Active(0.69) | Inactive(0.97) | Ⅴ(69.26%) |
| C8 | CC(C)N1C=NC2=C(N=C(N=C21)N(CCO)CCO)NCC3=CC=C(C=C3)OC | Inactive(0.85) | Inactive(0.51) | Ⅳ(67.38%) |
| C9 | C1CC1C2=CC(=NN2)NC3=NC(=NC=C3)NC4=CC5=C(C=C4)N=CN5 | Active(0.59) | Active(0.50) | Ⅴ(54.26%) |
| C10 | CC(C)CC(=O)NC1=NNC2=C1CN(C2(C)C)C(=O)C3CCN(CC3)C | Inactive(0.65) | Inactive(0.61) | Ⅳ(54.26%) |
| C11 | CC(C)C1=C2N=C(C=C(N2N=C1)NCC3=CC=CC=C3)NCCCCCCN.Cl | Inactive(0.69) | Active(0.54) | Ⅲ(54.26%) |
| C12 | CN1CCC(CC1)OC2=CC(=C(C=C2)NC3=NC=C4C(=N3)N(C(=O)N4C)C5CCCC5)OC | Inactive(0.72) | Inactive(0.54) | Ⅳ(67.38%) |
| C13 | CC(C)(C1=NC=C(C=C1)C2=CN=C3C(=N2)N(C(=O)CN3)C4CCC(CC4)OC)O | Inactive(0.65) | Inactive(0.65) | Ⅳ(54.26%) |
| C14 | C1CCN(CC1)CCOC2=CC=CC(=C2)C3=NNC4=C3C=C(C=C4)C5=NC=NN5 | Inactive(0.61) | Inactive(0.54) | Ⅳ(67.38%) |
| C15 | CCCCNC1=NC=C2C(=CN(C2=N1)C3CCC(CC3)O)C4=CC=C(C=C4)CN5CCN(CC5)C | Inactive(0.76) | Inactive(0.58) | Ⅳ(67.38%) |
| C16 | C1CN(CCN1)C(=O)C2=CC=C(C=C2)/C=C/C3=NNC4=CC=CC=C43 | Inactive(0.62) | Inactive(0.54) | Ⅳ(67.38%) |
| C17 | CCC1=C2N=C(C=C(N2N=C1)NCC3=C[N+](=CC=C3)[O-])N4CCCC[C@H]4CCO | Inactive(0.70) | Active(0.68) | Ⅳ(54.26%) |
| C18 | CC(C)N1C=NC2=C(N=C(N=C21)N3CCCC[C@H]3CCO)NCC4=NC5=C(N4)C=CC(=C5F)F | Inactive(0.66) | Inactive(0.64) | Ⅳ(67.38%) |
| C19 | CN1CC[C@H]([C@@H]1CO)C2=C(C=C(C3=C2OC(=CC3=O)C4=C(C=C(C=C4)C(F)(F)F)Cl)O)O | Inactive(0.78) | Inactive(0.64) | Ⅳ（63.78%） |
| C20 | CC(C)C1=C2N=C(N=C(N2N=C1)NCC3=CC=CC=C3N4C=CC=N4)OC5CCCNC5 | Inactive(0.66) | Inactive(0.54) | Ⅳ（54.26%） |
| C21 | CC1=CNC2=C1C(=NC=N2)N3CCC(CC3)(CN)C(=O)NC4=CC(=CC=C4)OC(=O)N(C)C | Inactive(0.71) | Inactive(0.58) | Ⅳ（54.26%） |
| C22 | CC(C)[C@H](CO)NC1=NC(=C2C(=N1)N(C=N2)C(C)C)NC3=CC(=CC=C3)Cl | Inactive(0.61) | Inactive(0.51) | Ⅲ（67.38%） |
| C23 | CCCN1C=NC2=C(N=C(N=C21)N[C@H](CC)CO)NCC3=CC=CC=C3 | Inactive(0.75) | Inactive(0.55) | Ⅲ（68.07%） |
| C24 | COC1=C(C=CC(=C1)N2CCC(CC2)N)NC3=NC=C(C(=N3)C4=CNC5=CC=CC=C54)Cl | Inactive(0.59) | Inactive(0.50) | Ⅲ（54.26%） |

**Notes:** Inactive indicates no significant toxic risk, while Active indicates potential toxic risk. The Probability value represents the model’s confidence level for the prediction result (values closer to 1 correspond to higher prediction reliability). Acute Oral Toxicity (per GHS criteria): Toxicity is classified into Grades I to VI (toxicity decreases sequentially). The Accuracy value quantifies the model’s prediction precision for acute oral toxicity grading.

**Table S4. DFT-calculated physicochemical parameters of hit compounds (C1–C23) and the positive control AZD-3463 (C24).**

| **S.no** | **SCF energy**  **(Hartree)** | **E_ZPE-corr_**  **(Hartree)** | **G_298K_**  **(Hartree)** | **Dipole**  **(D)** | **Imaginary frequency** | **HOMO**  **(eV)** | **LUMO**  **(eV)** | **E_gap_**  **(eV)** |
| --- | --- | --- | --- | --- | --- | --- | --- | --- |
| C1 | -953.7869 | -953.5669 | -953.6112 | 4.9828 | None | -6.6679 | -3.0893 | 3.5786 |
| C2 | -878.5636 | -878.3474 | -878.3900 | 2.6998 | None | -6.6322 | -3.1035 | 3.5288 |
| C3 | -993.0933 | -992.8447 | -992.8911 | 2.1753 | None | -6.4589 | -3.0175 | 3.4414 |
| C4 | -1526.7376 | -1526.2199 | -1526.2834 | 5.6427 | None | -1.9105 | -1.5894 | 0.3211 |
| C5 | -1143.4843 | -1143.0444 | -1143.1056 | 1.3824 | None | -5.3103 | -0.4890 | 4.8213 |
| C6 | -1028.9583 | -1028.7340 | -1028.7791 | 5.9341 | None | -6.1443 | -2.0779 | 4.0665 |
| C7 | -1104.1779 | -1103.9494 | -1103.9952 | 7.8261 | None | -6.0328 | -1.4389 | 4.5938 |
| C8 | -1333.2143 | -1332.7370 | -1332.8034 | 2.2715 | None | -5.3936 | -0.6789 | 4.7146 |
| C9 | -1095.4709 | -1095.1478 | -1095.1995 | 4.9468 | None | -5.4616 | -1.0335 | 4.4281 |
| C10 | -1167.4516 | -1166.9526 | -1167.0134 | 5.3361 | None | -1.3127 | -0.5448 | 0.7679 |
| C11 | -1186.6597 | -1186.1291 | -1186.1956 | 6.7922 | None | -2.0515 | -0.8052 | 1.2463 |
| C12 | -1488.5290 | -1488.0269 | -1488.0949 | 2.9037 | None | -1.5644 | -1.2174 | 0.3469 |
| C13 | -1315.9507 | -1315.5183 | -1315.5766 | 3.6452 | None | -6.0643 | -2.0384 | 4.0259 |
| C14 | -1257.0119 | -1256.5853 | -1256.6432 | 6.8453 | None | -5.6869 | -1.8327 | 3.8542 |
| C15 | -1495.4197 | -1494.7574 | -1494.8365 | 3.4783 | None | -1.5949 | -0.7510 | 0.8438 |
| C16 | -1068.9242 | -1068.5438 | -1068.5966 | 9.4636 | None | -2.2833 | -1.7794 | 0.5040 |
| C17 | -1296.0090 | -1295.5280 | -1295.5904 | 5.7971 | None | -5.0727 | -1.6817 | 3.3911 |
| C18 | -1606.3186 | -1605.8107 | -1605.8763 | 7.1060 | None | -5.1688 | -1.2683 | 3.9005 |
| C19 | -2040.9149 | -2040.5406 | -2040.6015 | 18.3987 | None | -2.5913 | -2.1698 | 0.4215 |
| C20 | -1407.0674 | -1406.5579 | -1406.6240 | 3.3155 | None | -1.9217 | -1.3306 | 0.5910 |
| C21 | -1503.4280 | -1502.9052 | -1502.9731 | 8.3416 | None | -1.9840 | -1.1353 | 0.8487 |
| C22 | -1603.080 | -1602.6497 | -1602.7115 | 4.7944 | None | -5.5002 | -1.0038 | 4.4964 |
| C23 | -1143.4793 | -1143.0389 | -1143.1006 | 1.7120 | None | -5.2951 | -0.5206 | 4.7745 |
| C24 | -1794.0781 | -1793.6045 | -1793.6659 | 1.4174 | None | -2.1709 | -1.5165 | 0.6544 |

**Table S5. ∆G_bind_(kcal/mol) of C18 with wild-type ALK, ALK mutants, and ten homologous tyrosine kinases, calculated from six independent 100 ns molecular dynamics simulations.**

| **Compound** | **Target Protein** | **PDB ID** | **∆G_bind_(kcal/mol)** |
| --- | --- | --- | --- |
| C18 | ALK(Wild-Type) | 4MKC | -41.32 ± 1.70 |
| C18 | ALK(F1174L) | 2YJR | -40.64 ± 4.38 |
| C18 | ALK(R1275Q) | 4FNY | -42.75 ± 0.84 |
| C18 | EGFR | 2GS2 | -36.72 ± 3.48 |
| C18 | RET | 2IVU | -38.45 ± 3.49 |
| C18 | ROS1 | 7Z5W | -36.93 ± 2.74 |
| C18 | MET | 5HTI | -39.38 ± 1.02 |
| C18 | KIT | 8PQA | -35.46 ± 4.31 |
| C18 | AXL | 5U6B | -38.10 ± 2.24 |
| C18 | TRKC | 3V5Q | -38.06 ± 1.47 |
| C18 | TRKB | 4AT5 | -39.56 ± 1.15 |
| C18 | TRKA | 5WR7 | -38.85 ± 5.08 |
| C18 | FGFR2 | 10OO | -39.07 ± 2.27 |

**Table S6. IC_50_ values of hit compounds against NB cell lines.**

| **NB cell line** | **Hit Compound** | **IC_50_ (μM)** | **95% CI** |
| --- | --- | --- | --- |
| **SH-SY5Y** | **C18** | 0.03666 | 0.03017 ~ 0.04464 |
| **SH-SY5Y** | **C17** | 0.05287 | 0.03040 ~ 0.09137 |
| **SH-SY5Y** | **C4** | 0.8800 | 0.6418 ~ 1.194 |
| **SH-SY5Y** | **C20** | 2.005 | 1.056 ~ 3.567 |
| **SH-SY5Y** | **C11** | 2.870 | 2.326 ~ 3.534 |
| **SH-SY5Y** | **C22** | 3.119 | 2.323~ 4.183 |
| **SH-SY5Y** | **C12** | 3.340 | 2.154 ~ 4.791 |
| **SH-SY5Y** | **C15** | 6.917 | 4.980 ~ 9.886 |
| **SH-SY5Y** | **C8** | 8.470 | 6.636 ~ 10.64 |
| **SH-SY5Y** | **C16** | 12.03 | 7.459 ~ 23.62 |
| **SH-SY5Y** | **C5** | 14.79 | 12.91 ~ 17.04 |
| **SH-SY5Y** | **AZD-3463** | 0.1002 | 0.08053 ~ 0.1238 |
| **SK-N-BE(2)** | **C4** | 0.1827 | 0.1361 ~ 0.2459 |
| **SK-N-BE(2)** | **C11** | 0.8073 | 0.3922 ~ 1.470 |
| **SK-N-BE(2)** | **C12** | 1.434 | 0.5398 ~ 3.602 |
| **SK-N-BE(2)** | **C17** | 0.01977 | 0.01431 ~ 0.02757 |
| **SK-N-BE(2)** | **C18** | 0.04655 | 0.02128 ~ 0.09917 |
| **SK-N-BE(2)** | **C20** | 0.6090 | 0.4147 ~ 0.8653 |
| **SK-N-BE(2)** | **C22** | 2.822 | 2.157~ 3.670 |
| **SK-N-BE(2)** | **AZD-3463** | 0.2500 | 0.1344 ~ 0.4750 |
| **IMR-32** | **C4** | 0.01431 | 0.01296 ~ 0.01580 |
| **IMR-32** | **C11** | 1.928 | 1.324 ~ 2.781 |
| **IMR-32** | **C12** | 1.691 | 1.029 ~ 2.706 |
| **IMR-32** | **C17** | 0.001973 | 0.001347 ~ 0.002670 |
| **IMR-32** | **C18** | 0.01275 | 0.01085 ~ 0.01497 |
| **IMR-32** | **C20** | 1.027 | 0.6973 ~ 1.490 |
| **IMR-32** | **C22** | 0.8741 | 0.6766~ 1.122 |
| **IMR-32** | **AZD-3463** | 1.282 | 0.9352 ~ 1.748 |
